# A New Approach for the Study of Lung Smooth Muscle Phenotypes and Its Application in a Murine Model of Allergic Airway Inflammation

**DOI:** 10.1371/journal.pone.0074469

**Published:** 2013-09-09

**Authors:** Jesus Paez-Cortez, Ramaswamy Krishnan, Anneliese Arno, Linh Aven, Sumati Ram-Mohan, Kruti R. Patel, Jining Lu, Oliver D. King, Xingbin Ai, Alan Fine

**Affiliations:** 1 The Pulmonary Center, Boston University School of Medicine, Boston, Massachusetts, United States of America; 2 Center for Vascular Biology Research, Beth Israel Deaconess Medical Center, Boston, Massachusetts, United States of America; 3 Department of Cell and Developmental Biology, University of Massachusetts Medical School, Worcester, Massachusetts, United States of America; Children’s Hospital Los Angeles, United States of America

## Abstract

Phenotypes of lung smooth muscle cells in health and disease are poorly characterized. This is due, in part, to a lack of methodologies that allow for the independent and direct isolation of bronchial smooth muscle cells (BSMCs) and vascular smooth muscle cells (VSMCs) from the lung. In this paper, we describe the development of a bi-fluorescent mouse that permits purification of these two cell populations by cell sorting. By subjecting this mouse to an acute allergen based-model of airway inflammation that exhibits many features of asthma, we utilized this tool to characterize the phenotype of so-called asthmatic BSMCs. First, we examined the biophysical properties of single BSMCs from allergen sensitized mice and found increases in basal tone and cell size that were sustained *ex vivo*. We then generated for the first time, a comprehensive characterization of the global gene expression changes in BSMCs isolated from the bi-fluorescent mice with allergic airway inflammation. Using statistical methods and pathway analysis, we identified a number of differentially expressed mRNAs in BSMCs from allergen sensitized mice that code for key candidate proteins underlying changes in matrix formation, contractility, and immune responses. Ultimately, this tool will provide direction and guidance for the logical development of new markers and approaches for studying human lung smooth muscle.

## Introduction

Despite their structural and functional importance in the airway and pulmonary vasculature, defining features for bronchial smooth muscle (BSM) and vascular smooth muscle (VSM) in developing, mature, and diseased lungs have been poorly characterized. These gaps in knowledge are largely due to the lack of methodologies that allow direct and high fidelity isolation of bronchial smooth muscle cells (BSMCs) and vascular smooth muscle cells (VSMCs). Due to the absence of such methodologies, our current understanding of the lung smooth muscle phenotype is primarily derived from the study of *in vitro* cultured and passaged cells, and from the morphological and histological study of lung tissue [1]–[4]. In view of the limitations of these approaches, the precise pathophysiological differences in global gene expression, cell size, and tone between normal and diseased smooth muscle cells remain unresolved [5]. Indeed, the shortcomings of mouse models of airway and pulmonary vascular disease are attributable, in part, to the limited tools available to purify and analyze lung smooth muscle populations. As a consequence, the development of meaningful approaches for isolation and comprehensive analysis of human lung smooth muscle cells has stagnated. From a clinical standpoint, this state-of-affairs is one of the key factors that underlie the lack of treatments aimed at reversing the pathological smooth muscle phenotypes characteristic of diseases such as asthma and pulmonary hypertension [6]–[8].

To further the study of lung smooth muscle phenotypes, we sought to develop a methodology that allows the selective and independent isolation of BSMCs or VSMCs from the lungs of control and diseased mice. To do this, we generated a bi-fluorescent transgenic mouse in which BSMCs singly express a green fluorescent protein (hrGFP) whereas VSMCs express both a green (hrGFP) and a red fluorescent protein (DsRed). From this mouse, cell populations that are highly enriched for BSMCs or VSMCs can be independently collected by cell sorting for further characterization. This methodology thus provides a tool to directly study smooth muscle cell populations in mouse models of lung disease.

As a proof-of-concept, we used this methodology to characterize biophysical and gene expression properties of BSMCs in an allergen model of airway inflammation. For this, we purified BSMC from bi-fluorescent mice subjected to ovalbumin (OVA) sensitization and challenge to induce acute allergic inflammation. We employed this model because it displays many of the salient features of human asthma, including airway hyper-reactivity. The ability to isolate individual smooth muscle cells allowed us to demonstrate that single BSMCs from mice subjected to OVA-induced airway inflammation exhibit increased cell size and cellular contractile tone, as compared to cells isolated from control mice that were sensitized with PBS saline. By performing comprehensive profiling of isolated RNA followed by gene set analysis, we went on to identify expression changes in multiple gene categories and signaling pathways in BSMCs from OVA sensitized mice. Together, these findings provide for the first time an expansive and integrated delineation of a putative acute asthmatic BSMC phenotype. By doing so, we demonstrate the power of this unique methodology for addressing fundamental issues in lung smooth muscle biology.

## Results

### Development of a Bi-fluorescent Mouse for Lung Smooth Muscle Isolation

**Figure 1:**
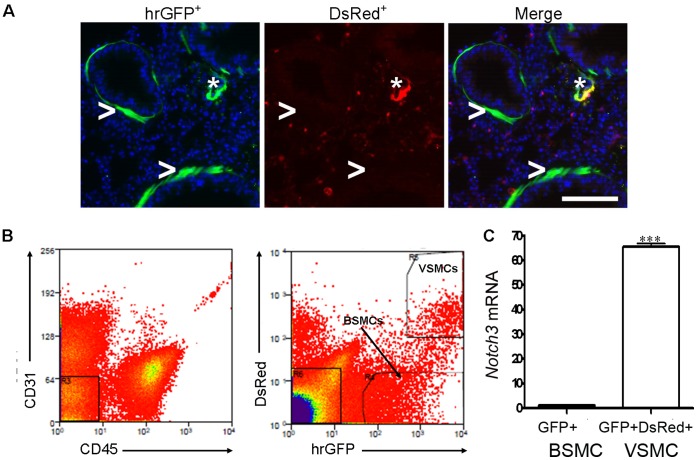
Separation of BSMCs and VSMCs from the lung using the bi-fluorescent *αSMA-hrGFP;NG2-DsRed* mouse. (**A**) Expression pattern of hrGFP and DsRed in BSMCs and VSMCs in the lung of an *αSMA-hrGFP;NG2-DsRed* mouse. Both BSMCs and VSMCs are hrGFP^+^. BSMCs are DsRed^−^ whereas VSMCs are DsRed^+^. Asterisk (*) indicates the blood vessel in the lung the airway. Arrows (>) indicate the airway. Scale bar: 20 µm. (**B**) Algorithm for bronchial and vascular smooth muscle cell identification by flow cytometry. CD31^+^ endothelial cells and CD45^+^ immune cells were separated from dissociated lung preparation (left panel) followed by evaluation of hrGFP and DsRed distribution within CD31^−^CD45^−^ population (middle panel). CD31^−^CD45^−^hrGFP^+^DsRed^−^ population corresponds to BSMCs (pointed by an arrow). CD45^−^CD31^−^hrGFP^+^DsRed^+^ population is enriched in vascular smooth muscle cells (VSMCs). (**C**) Relative *Notch3* mRNA expression in isolated singly hrGFP^+^ cells and doubly hrGFP^+^DsRed^+^ cells from lung cell preparation as determined by qPCR followed by normalization to 18S. Data show mean ± SD and are representative of three independent experiments. ***p<0.001.

We previously generated a transgenic mouse (*αSMA-hrGFP*) in which hrGFP is expressed under the control of the *α-smooth muscle actin (SMA)* gene promoter [9]. In this mouse, hrGFP is selectively expressed in BSMCs and VSMCs (Fig. 1A). To allow further separation of these two smooth muscle populations, we crossed the *αSMA-hrGFP* mouse with a transgenic *NG2-DsRed* mouse [10]. In this double transgenic mouse, the DsRed fluorescent protein is expressed in VSMCs, pericytes, and some macrophages (Fig. 1A). Thus, in the bi-fluorescent transgenic (*αSMA-hrGFP;NG2-DsRed*) mouse, hrGFP is singly expressed in BSMCs whereas hrGFP and DsRed are doubly expressed in VSMCs (Fig. 1A); thereby allowing the independent isolation of BSMCs and VSMC from the lung by cell sorting.

To maximize smooth muscle purity, we designed a cell sorting algorithm through which singly hrGFP^+^ cells or doubly hrGFP^+^DsRed^+^ were collected from lung cell preparations devoid of endothelial and hematopoietic lineages (CD31^−^CD45^−^) (Fig. 1B). From a single proteolytically dissociated adult mouse lung, 0.4%–0.9% of total cells are singly hrGFP^+^ and 0.04%–0.1% are doubly hrGFP^+^DsRed^+^ cells (data not shown). As predicted, these two cell populations express high levels of *αSMA* mRNA relative to hrGFP^−^DsRed^−^ lung cells (data not shown). Notably, no hrGFP^+^ or DsRed^+^ cells were identified by cell sorting in the lungs of wild type mice that do not express these fluorescent genes (Figure S1 in File S1). In addition, post-sort cell purity tests showed that approximately 95% of sorted populations express appropriate BSMC and VSMC markers (Figures S2, S3 in File S1). To further validate the identity of singly hrGFP^+^ and doubly hrGFP^+^DsRed^+^ fluorescent cells, relative *Notch3* mRNA levels were examined since we previously found that Notch3 expression is restricted to VSMCs in the lung [9]. As shown, singly hrGFP^+^ cells express *Notch3* mRNA at a level approximately 70-fold lower than hrGFP^+^DsRed^+^ cells (Fig. 1C). Together, these findings indicate that we have established a methodology for the isolation of highly enriched populations of BSMCs and VSMCs.

### A model of Acute Allergic Asthma using the Bi-fluorescent Mouse

**Figure 2:**
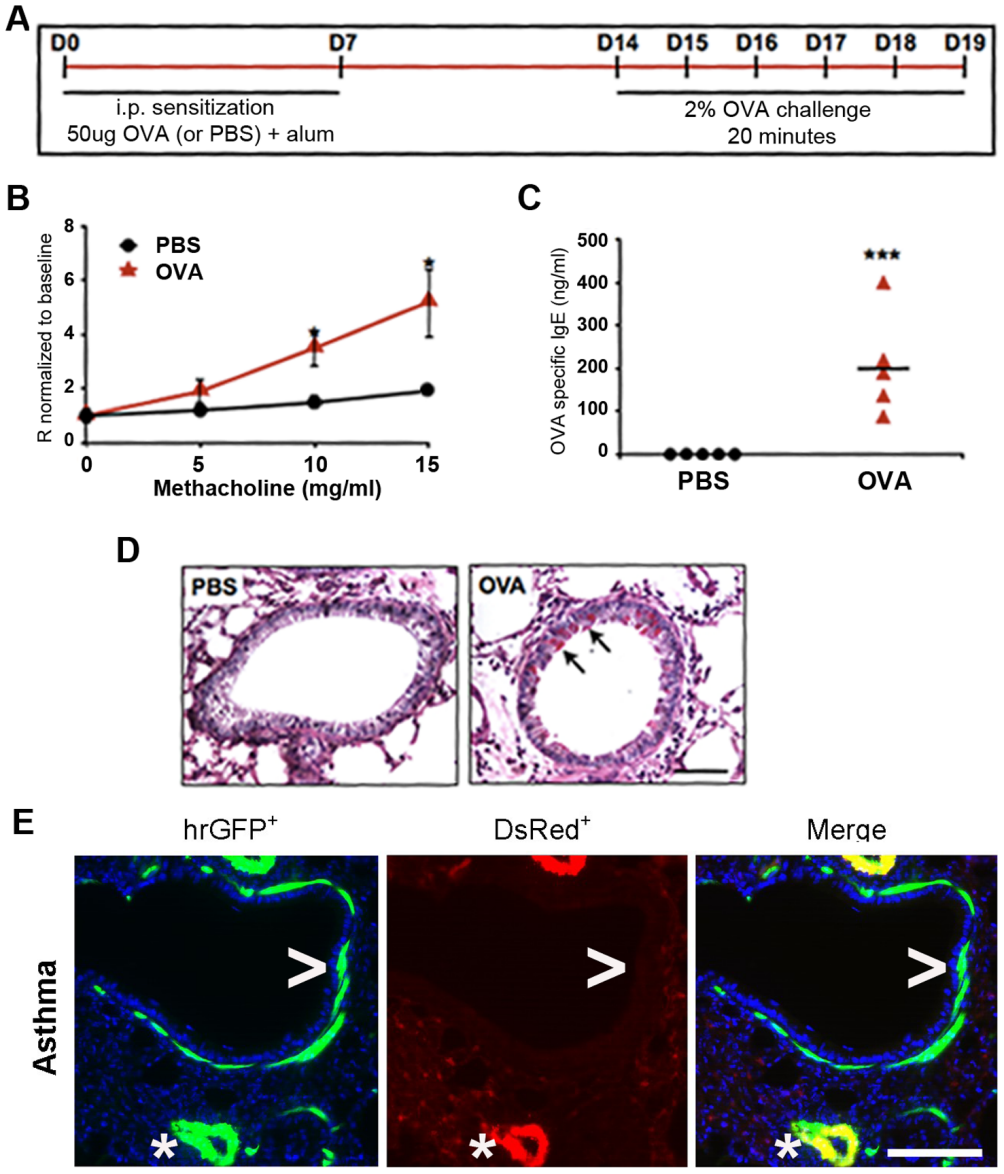
Characterization of an allergen sensitized mouse model of asthma using *αSMA-hrGFP;NG2-DsRed* mouse. (**A**) A protocol to induce acute allergic airway inflammation using OVA sensitization and challenge. Mice sensitized with saline were control. (**B**) Airway reactivity in response to increasing doses of Mch in control (•) and OVA-sensitized (▴) mice by Flexivent. The measurements represented Rn values. (**C**) Serum levels of OVA-specific IgE in control (▪) and OVA-sensitized (•) mice, as measured by ELISA. (**D**) PAS staining for mucin production in the lung epithelium of control and OVA-sensitized mice. (**E**) The expression of hrGFP and DsRed in the lungs of OVA-sensitized mice. Scale bar, 20 µm. *P<0.05.

Taking advantage of this methodology, we sought to identify the phenotype of so-called asthmatic BSMCs from OVA sensitized mice. To do this, acute allergic airway inflammation was induced in *αSMA-hrGFP;NG2-DsRed* mice by OVA sensitization and challenge followed by BSMC isolation (Fig. 2A) [11]. The induction of an asthma-like state in these mice was confirmed by hyper-reactivity to methacholine measured by Flexivent, elevated serum levels of OVA-specific IgE, and increased mucus production in the airway epithelium, as compared to control mice that were OVA challenged but sensitized with PBS (Fig. 2B–D). Importantly, the cell specific expression pattern of hrGFP and DsRed is unchanged after OVA sensitization and challenge (Fig. 2E), substantiating the use of the sorting algorithm for isolating BSMCs. In this regard, we did not detect hrGFP positivity in any cells outside of the smooth muscle compartments after allergen challenge by fluorescent microscopy (Fig. 2E). Further, we only collected cells with relatively high levels of hrGFP expression (Fig. 1B), thereby avoiding the potential contamination of myofibroblast cells that are known to express low levels of αSMA [12].

### Physical Properties of Single BSMCs from Sensitized Mice

**Figure 3:**
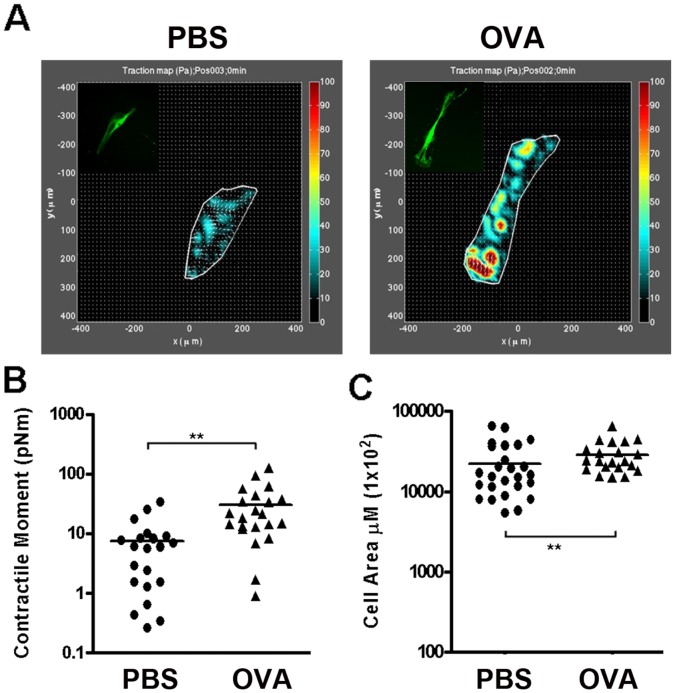
Measurement of changes in physical properties of individual BSMCs from OVA sensitized mice. BSMCs isolated from PBS-sensitized control and OVA sensitized *αSMA-hrGFP;NG2-DsRed* mice were allowed to attach to collagen I gel in culture for 72 hrs before assays. (**A**) Images of hrGFP^+^ BSMCs from PBS control and OVA sensitized mice (insert) and their traction maps on collagen I gels. (**B**) Contractile moment measurement of individual BSMCs from control and sensitized mice based on their traction maps. The line represents the mean of individual measurements of control (n = 20) and acute asthmatic (n = 22) cells. (**C**) Measurement of cell size of BSMCs from PBS sensitized control and OVA sensitized mice. The line represents the mean of individual measurements. **p<0.008.

The methodology described herein allowed us to examine whether individual cells derived from airways with allergen-induced airway inflammation display measurable differences in contractile tone and cell size. To address this question, we employed an established cellular force measurement technology called traction force microscopy [13]. For this, cells were cultured sparsely on a deformable gel substrate (Youngs Modulus = 4 kPa). Embedded in the substrate were fiducial markers consisting of fluorescent nanobeads. By tracking displacement of the beads and solving the inverse problem of forces necessary to induce those displacements, we quantified traction, the contractile forces that cells exert on their substrate (Fig. 3). From the traction map, we extracted a single measure of overall cell contractility, which is referred to as the net contractile moment (Fig. 3A). By this method, we determined that single BSMCs from OVA sensitized mice had a four-fold greater contractile moment than normal PBS sensitized cells, indicating increased basal tone (Fig. 3B). These changes were also associated with significant enhancements in cell spreading area, consistent with the increase in cell size in BSMCs from sensitized mice (Fig. 3C).

### Gene Expression Analysis of BSMCs Isolated from the Mouse Model of Acute Asthma

Differences in the biophysical properties of isolated BSMCs from OVA sensitized mice demonstrate that so-called asthmatic BSMCs have a distinctly altered phenotype that is maintained after isolation. In order to further clarify this phenotype, we used Affymetrix^R^ whole-transcript microarrays to assess comprehensive mRNA levels in BSMCs isolated from OVA sensitized and PBS sensitized control bi-fluorescent mice. Of the 20,719 mouse genes with Entrez IDs represented on the arrays, 443 showed a difference between OVA and PBS arrays with p-value<0.01, including 240 genes down-regulated and 203 genes up-regulated in OVA samples (Table S2 in File S1). This cutoff had an associated false-discovery rate (FDR) of 0.47. Controlling for FDR<0.25 by using a more stringent cutoff (nominal p-value<0.0002) resulted in 14 genes differentially expressed, 4 down-regulated and 10 up-regulated in OVA samples (Table 1).

**Table 1:** Genes differentially expressed between BSMCs from PBS sensitized control and OVA sensitized mice.

Symbol	Entrez ID	Gene name	LFC	*p*-value	FDR
*Adh7*	11529	alcohol dehydrogenase 7 (class IV), mu or sigma polypeptide	−1.04	4.9e-05	0.23
*Cyp2f2*	13107	cytochrome P450, family 2, subfamily f, polypeptide 2	−1.04	9.5e-05	0.23
*Gabrb1*	14400	gamma-aminobutyric acid (GABA) A receptor, subunit beta 1	−0.96	1.4e-04	0.24
*Gabra4*	14397	gamma-aminobutyric acid (GABA) A receptor, subunit alpha 4	−0.78	8.1e-05	0.23
*Postn*	50706	periostin, osteoblast specific factor	0.67	1.6e-04	0.24
*Retnlg*	245195	resistin like gamma	1.01	1.0e-04	0.23
*Slc26a4*	23985	solute carrier family 26, member 4	1.41	7.0e-05	0.23
*Egln3*	112407	EGL nine homolog 3 (C. elegans)	1.88	1.6e-04	0.24
*Cfi*	12630	complement component factor i	2.05	3.4e-06	0.07
*Il13ra2*	16165	interleukin 13 receptor, alpha 2	2.11	2.1e-05	0.22
*Cysltr1*	58861	cysteinyl leukotriene receptor 1	2.13	7.9e-05	0.23
*Fbp1*	14121	fructose bisphosphatase 1	2.22	3.2e-05	0.22
*Tdo2*	56720	tryptophan 2,3-dioxygenase	2.33	1.1e-04	0.23
*Col6a5*	665033	collagen, type VI, alpha 5	3.50	1.6e-04	0.24

Genes with significant expression differences at false-discovery rate (FDR)<0.25, ordered by increasing log_2_ fold-change (LFC). Negative LFC indicates down-regulation in OVA sensitized samples, and positive LFC indicates up-regulation. The first column gives the Affymetrix MoGene-1_0-st-v1.r4 probe set ID for the gene.

We then used the gene set analysis method CAMERA [14] to look for biologically relevant groups of genes that collectively showed significant expression differences (down or up) while adjusting for inter-gene correlation. Table 2 shows the most significantly down- and up-regulated gene sets among the MSigDB [15] canonical pathways, Gene Ontology categories, and motif gene sets (based on transcription factor or micro-RNA binding sites). Down-regulated gene sets included the ligand gated ion channel transport pathway, the transforming growth factor beta receptor signaling pathway, and the motif set V$E4BP4_01 consisting of genes whose promoter region contains the binding motif of E4BP4, also known as NFIL3 (nuclear factor, interleukin 3 regulated). Up-regulated gene sets included the platelet-derived growth factor (PDGF) pathway, the lymphangiogenesis pathway, and the SH2 domain binding category.

**Table 2:** Gene sets misregulated in BSMCs from OVA sensitized mice as compared to PBS sensitized control.

MSigDB gene set	Size	*p*-value	Biological Function
**Canonical pathways, down in asthma vs. control arrays**			
REACTOME_LIGAND_GATED_ION_CHANNEL_TRANSPORT	19	0.001	Ion channels that respond to chemical messenger biding
MIPS_P2X7_RECEPTOR_SIGNALLING_COMPLEX	12	0.001	Ion transport, ion channel activity, cell surface receptor linked signal transduction, cytoskeleton organization and biogenesis
REACTOME_TIE2_SIGNALING	17	0.001	vascular and hematopoietic development
REACTOME_PI3K_AKT_ACTIVATION	35	0.001	Control of smooth muscle tone via cAMP signaling pathways
REACTOME_SIGNALING_BY_FGFR_MUTANTS	43	0.003	cell fate and differentiation
**Canonical pathways, up in asthma vs. control arrays**			
BIOCARTA_PDGF_PATHWAY	32	0.005	cellular proliferation and development
MIPS_ARC92_MEDIATOR_COMPLEX	12	0.011	transcriptional activator activity
PID_LYMPHANGIOGENESIS_PATHWAY	25	0.016	
MIPS_ARC_L_COMPLEX	14	0.019	regulation of transcription factor activity
BIOCARTA_RAC1_PATHWAY	23	0.021	Cell motility regulation
**Gene Ontology categories, down in asthma vs. control arrays**			
TRANSFORMING_GROWTH_FACTOR_BETA_RECEPTOR_SIGNALING_PATHWAY	36	0.001	Cell growth and Apoptosis
PERIPHERAL_NERVOUS_SYSTEM_DEVELOPMENT	12	0.002	Peripheral Neuronal development
AXONOGENESIS	42	0.003	Growth and differentiation of axons
TRANSMEMBRANE_RECEPTOR_PROTEIN_SERINE_THREONINE_KINASE_SIGNALING_PATHWAY	47	0.004	Cellular signaling, gene transcription
NEGATIVE_REGULATION_OF_CELL_DIFFERENTIATION	27	0.005	Reduction or prevention of cell differentiation
**Gene Ontology categories, up in asthma vs. control arrays**			
MRNA_SPLICE_SITE_SELECTION	13	0.006	Spliceosomal complex formation and biosynthesis
SH2_DOMAIN_BINDING	15	0.007	Recognigtion of the phosphorylated state of tyrosine residues
SPINDLE_MICROTUBULE	16	0.018	Regulation of microtubule that is part of a mitotic or meiotic spindle
NLS_BEARING_SUBSTRATE_IMPORT_INTO_NUCLEUS	12	0.018	Translocation of protein bearing a nuclear localization signal (NLS) from the cytoplasm into the nucleus
STEROID_HORMONE_RECEPTOR_BINDING	10	0.021	Steroid hormone regulation
**Motif gene sets, down in asthma vs. control arrays**			
TACGGGT,MIR-99A,MIR-100,MIR-99B	23	0.002	Matrix associated genes
V$E4BP4_01	216	0.002	Immune cell differentiation and activation
V$EN1_01	107	0.003	Cell development and differentiation
V$FOXD3_01	191	0.003	Transcriptional repression
V$HOXA3_01	13	0.006	gene expression, morphogenesis, and differentiation

The five most significantly down- and up-regulated gene sets among the MSigDB canonical pathways, Gene Ontology categories, and motif gene sets are shown, ordered in each subsection by CAMERA *p*-value. No motif gene set was up-regulated with p<0.05, so these are excluded. The size column gives the number of genes in the gene set after restricting to members with mouse orthologs represented by probe sets on the arrays.

### Validation of Array Findings

**Figure 4:**
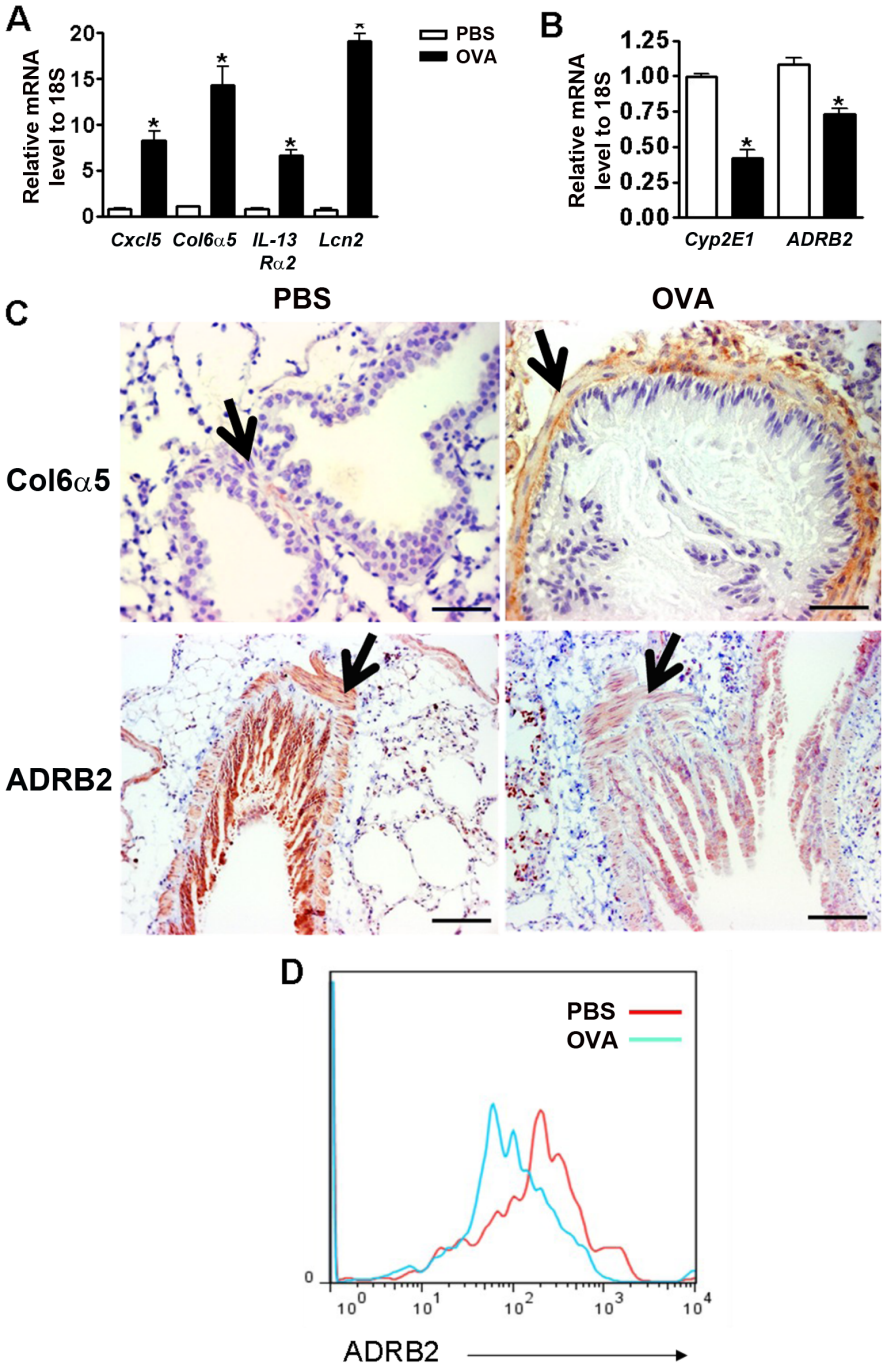
Validation of selected deregulated genes in BSMCs from OVA sensitized mice identified by the array. (**A, B**) Real time PCR validation of selected up-regulated genes (**A**) and down-regulated genes (**B**) genes in BSMCs from OVA sensitized mice. Results were normalized to 18S. Data show mean ± SD and are representative of three independent experiments. *p<0.05. (**C**) Immuno-histochemistry for collagen 6 alpha 5 (COL6A5) and the adrenergic β2-receptor (ADRB2) in lungs of PBS sensitized control and OVA sensitized mice. Lung sections of control and sensitized mice were stained side-by-side under the same condition. Black arrows indicate BSMC layer. Scale bar: 20 µm. (**D**) Flow cytometry for the ADRB2 receptor in non-permeabilized BSMCs from PBS sensitized control (red) and OVA sensitized (blue) mice. Data is representative of two independent experiments.

We selected a group of genes deregulated in BSMCs from OVA sensitized mice for validation by qPCR and immunohistochemistry. This group contains genes that displayed either a high fold-change in expression (*lipocalin 2 (Lcn2)*, *Cxcl5*, *Cyp2eE1*), an FDR<0.25 (*Col6α5*, *IL13Rα2*), or a direct functional relevance to airway physiology (*adrenergic receptor β2* (*Adrb2)* [16]. Consistent with the array data, the mRNA expression of *Cxcl5*, *Col6α5*, *IL13Rα2*, and *Lcn2* genes were significantly up-regulated in BSMCs from OVA sensitized mice, and the mRNA expression of cytochrome P45 family gene *Cyp2eE1*, and *Adrb2* were significantly down-regulated as compared to controls (Fig. 4A, 4B). Also, in accordance with the array, immuno-staining of lung sections showed that Col6α5 protein level was increased in BSMCs from OVA sensitized mice while the adrenergic receptor β2 was decreased in BSMCs and interestingly also in the overlying epithelium of OVA sensitized mice (Fig. 4C). Notably, immunostaining of lung sections without primary antibodies yielded no signal. Since we could directly isolate pure BSMCs, we interrogated changes in adrenergic receptor β2 cell surface expression in BSMCs from OVA sensitized mice by flow cytometry. This analysis demonstrated that down-regulation of *Adrb2* mRNA is associated with a decrease in the number of receptors on the cell surface of BSMCs from OVA sensitized mice (Fig. 4D). Most notably, the arrays showed that there were no significant differences in the mRNA expression for any acetylcholine receptors, either nicotinic (Gene Ontology category GO:0004889) or muscarinic (GO:0016907).

## Discussion

Despite its fundamental importance to the pathogenesis and clinical manifestations of diseases of the airway and pulmonary circulation, an understanding of the integrated phenotypes that define diseased smooth muscle has remained elusive [4]. This ongoing issue reflects the lack of tools that support a direct isolation of BSMCs and VSMCs for analysis. To address this, we successfully developed a mouse-based tool that sustains the independent isolation of BSMCs and VSMCs for detailed characterization. This tool can now be used to address key questions related to the biology of lung smooth muscle during development, homeostasis, and disease conditions. Using this methodology, the transcriptional, post-transcriptional, and epigenetic mechanisms that govern global gene expression in smooth muscle can be examined.

This approach afforded us the opportunity to demonstrate that measurable increases in contractile tone characterize individual BSMCs from sensitized mice, which we hope will help lay to rest this controversial issue [3]. We also generated the first comprehensive characterization of the global gene expression changes that define a putative acute asthmatic BSMC phenotype. Collectively, these types of studies may provide direction and guidance for the logical development of new markers and approaches for studying human lung smooth muscle. We recognize that cell sorting procedures may affect gene expression. However, this potential limitation may accompany any method for RNA isolation including laser capture microdissection. In this work we identified changes in multiple genes, and through gene set analysis, identified several deregulated signaling pathways in BSMCs from an allergen sensitized mouse model of acute asthma (Table 2). Additionally, we observed that in a chronic model of allergen-induced airway inflammation in mice there is also a persistent down-regulation of the adrenergic receptor β2 (not shown) along with an additional increase in BSMC contractile tone and cell size (Figure S4 in File S1). These data suggest that the BSMC phenotype in persistent asthma is, in part, an enhancement of the acute phenotype. We recognize that some elements of the gene expression changes observed in BSMCs may be specific for this particular asthma mouse model [17]. Additional studies using the methodology herein will be required to evaluate which identified mRNA changes are model-specific and which are universal. It is also important to note that human asthma similarly comprises multiple clinical phenotypes [18]. Findings based on gene expression and pathway analysis using mouse BSMCs from allergen sensitized mice may help identify markers and key defects in human asthmatics.

Pathway analysis using CAMERA [14] identified an enrichment of misregulated genes in several distinct signaling pathways that can be further evaluated. We also employed the self-contained gene set analysis method ROAST [19] to assess the concordance of our array results with sets of genes identified as differentially expressed in previous asthma-related studies (Figure S5 in File S1). These previous studies largely involve analysis of RNA changes in whole lung or in cultured cells [3], [4], [20]. Of the 26 gene sets from 9 studies examined, 13 showed weak evidence of altered regulation (p<0.05) in our arrays, typically with no more than one or two genes individually passing the p<0.01 cutoff. The most significant agreement was seen for the set of genes induced by IL-4 and IL-13 in cultured human bronchial epithelial cells [20] (ROAST p-value for up-regulation 0.009), and for the set of genes inhibited by IL-13 in human airway epithelial cells [3] (ROAST p-value for down-regulation 0.008) (Figure S5 in File S1) thereby suggesting a common Th2 cytokine-dependent gene signature across cell types in asthma. In addition, we observed an up-regulation of the Rac1 pathway in BSMCs from sensitized mice, which may, relate, in part, to changes in tone.

Interestingly, we observed a reduction in cell surface receptors that mediate smooth muscle relaxation (β2 adrenergic and GABA), but did not detect any change in mRNAs for receptors that mediate bronchoconstriction (muscarinic and nicotinic) [16], [21]. We speculate, therefore, that increased methacholine reactivity in asthma is attributable, at least, in part, to diminished input of signals that relax smooth muscle. Together, these studies show how coupling gene expression data from isolated lung smooth muscle cell populations to physiological assays can potentially elucidate the molecular basis for key pathophysiological properties of a diseased airway or vessel.

In summary, the development of the bi-fluorescent mouse and the capacity to isolate smooth muscle populations from the lung opens opportunities to study the biology of lung smooth muscle. This is particularly relevant since alterations in smooth muscle phenotype are a prominent and defining feature in diseases of the airway and pulmonary vasculature. In addition, our studies of BSMCs from an asthma model provide proof-of-principle evidence that this methodology, coupled with disease models, provides an invaluable and versatile tool for characterization of the signals and pathways involved in the initiation and maintenance of abnormal smooth muscle phenotypes [5], [8].

## Materials and Methods

### Mice and a Mouse Model of Acute Allergic Airway Inflammation

A transgenic mouse (*αSMA-hrGFP*) expressing hrGFP under the control of the rat α-smooth muscle actin (αSMA) gene promoter was generated [9]. *αSMA-hrGFP* mice selectively express hrGFP in smooth muscle cell populations *in vivo*. C57/Bl6 and *NG2-DsRed* transgenic mice that expresses the red fluorescent protein (DsRed.T1) under the control of the mouse *NG2* (*Cspg4*) promoter [10] were obtained from Jackson Laboratories (Bar Harbor, MN). The *αSMA-hrGFP;NG2-DsRed* mouse was generated by crossing *αSMA-hrGFP* with *NG2-DsRed* mice. To induce acute allergic airway inflammation, adult mice received two intraperitoneal (i.p.) injections of 50 µg ovalbumin (OVA) in Imject Alum (Thermo Scientific, Waltham, MA) on days 0 and 7, followed by 20-minute nebulizations with a 2% OVA solution on days 14, 15, 16, 17, and 18. Control mice were sensitized with PBS followed by 5 nebulizations with OVA solution. Mice were analyzed on day 19. Description of this model is included in the supplementary material section. All animal studies were carried out in strict accordance with the recommendations in the Guide for the Care and Use of Laboratory Animals of the NIH. The protocol was approved by the Boston University IACUC Committee (protocol number: AN-15112).

### Assessment of Key Features of Asthma in Sensitized Mice

Airway resistance (Rn) was determined using a FlexVent ventilator (Scireq Scientific Respiratory Equipment. Montreal, Canada) at tidal volume of 6–7 ml/kg, a positive-end expiratory pressure of 3 cm H_2_O, and a respiratory rate of 190 breaths/minute. Baseline airway resistance was measured after airway delivery of nebulized vehicle, and similar measurements were performed at increasing concentrations of nebulized methacholine (Mch (5 mg/ml, 10 mg/ml, 15 mg/ml). Data are presented as normalized to baseline level. After final measurements, the mouse was disconnected from the ventilator for organ and body fluid harvest. Mouse OVA-specific IgE plasma levels were determined using a commercial ELISA kit (MdBioProducts. St Paul, MN).

### Lung Cell Isolation, Cell Sorting and Flow Cytometry

Cell suspensions were obtained by digesting *αSMA-hrGFP;NG2-DsRed* mouse lungs with a commercially available Lung Dissociation Kit (Miltenyi Biotech, Auburn, CA). Cells were further stained with anti-mouse CD45 (30-F11) and CD31 (MEC 13.3) antibodies (BD Pharmingen, San Diego, CA). Isotype antibodies were used as controls. BSMCs (CD45^−^CD31^−^hrGFP^+^NG2DsRed^−^) and VSMCs (CD45^−^CD31^−^hrGFP^+^NG2DsRed^+^) were collected using a Moflo cell sorter (Beckman Coulter, Fullerton, CA). For flow cytometry, cells were stained with fluorochrome-conjugated mouse-specific mAbs prior to analysis on a LSRII cytometer (BD Biosciences), and data were analyzed using FlowJo software (Tree Star Inc., Ashland, OR).

### Single Cell Contractile Moment Determination

The innate contractile phenotype of BSMCs *in vitro* was assessed by traction force microscopy [6], [13]. Briefly, isolated BSMCs cells were cultured on collagen-1 coated polyacrylamide gel substrates (stiffness 4 kPa) previously embedded with fluorescent nanobeads. After 72 hrs, cells were attached and subjected to measurements. The nanobeads serve as fiducial markers of the cell-exerted displacements. Based on the displacement field and the intrinsic stiffness of the substrate, the cells’ net contractile moment was computed using MATLAB.

### Immunohistochemistry and Immunofluorescence

Lungs were fixed with 4% paraformaldehyde, paraffin embedded, sectioned, deparaffinized, and subjected to antigen retrieval prior to primary and secondary antibody incubation. A biotinylated anti-α-smooth muscle actin antibody (1A4), an anti-mouse collagen 6A1, and an anti-mouse adrenergic receptor β2 antibody were purchased from Thermo Scientific (Waltham, MA), Proteintech (Chicago, IL) and Abcam (Cambridge, MA) respectively. Mucin production and airway inflammation were determined by Periodic acid-schiff (PAS) and Hematoxilin Eosin (HE) (Sigma Aldrich. St. Louis, MO) stainings, respectively.

### BSMC mRNA Profiling

Total RNA from purified BSMCs was isolated using the RNeasy mini-kit from Qiagen (Qiagen, Valencia, CA). Because of limited amount of BSMC RNA per mouse, each RNA sample for the array study was pooled from 5–8 mice [22]. Three controls and three asthmatic samples were subjected to mRNA profiling using the Affymetrix^R^ Mouse Gene 1.0 ST Whole Transcript platform at the Boston University Microarray Resource. Due to technical issues with one asthma sample, this array was an outlier based on principal components analysis and therefore was excluded from the analysis. Microarray background correction, normalization and probeset summarization were performed with the fitProbeLevelModel function in the R package oligo [23]. Probe sets with no associated Entrez gene ID were filtered out, and when multiple probe sets had the same Entrez ID, only the one with the largest intra-quartile range across all samples was retained. Differential expression between asthmatic and control arrays was assessed for the resulting 20,719 probe sets with the R package limma using *p*-values based on empirical-Bayes moderated *t*-statistics [24]. The false-discovery rate (FDR) [25] was used to adjust for multiple hypothesis testing. The limma function roast [19] was used for self-contained gene set tests of select lists of genes reported as differentially expressed in earlier asthma-related studies, and the limma function camera [14] was used for competitive tests of all gene-sets from MSigDB [15] (v3.1; http://www.broadinstitute.org/gsea/msigdb) with gene IDs converted using the table of human/mouse orthologs from MGI (ftp://ftp.informatics.jax.org/pub/reports/). The microarray data have been deposited at GEO (http://www.ncbi.nlm.nih.gov/geo/) with accession number GSE45723.

### Real Time PCR Analysis

BSMC RNA from control and asthmatic mice was isolated using a RNAeasy Mini kit (Qiagen, Valencia, CA). cDNA was transcribed using the Promega Reverse Transcription System (Promega, Madison, WI). qPCR was performed in a StepOne Plus instrument (Applied Biosystems, Foster City, CA). Reactions were normalized to 18s rRNA. The relative gene expression was measured by normalizing to 18S RNA using ΔCt (cycle threshold difference). The numbers were then multiplied by an artificial unit to make the value of control samples between 1 and 10 before presentation in figures. qPCR was performed with fast SYBR reagent (Invitrogen, CA) or using Taqman^R^ probes (Invitrogen, CA). See Table S1 in File S1 for primers details.

### Statistical Analysis

Statistical analysis of microarray data is described in the section “BSMC mRNA profiling”. Other data are presented as mean +/− standard deviation (SD), with statistical analysis performed using Student’s t test. Significant differences are indicated by *p<0.05, **p<0.01, ***p<0.001.

## Supporting Information

File S1 **All supporting materials, including supplementary methods, tables, and figures are included in File S1.** Table S1 lists all primer sequences for real-time PCR. Table S2 lists all deregulated gene list in BSMCs from OVA sensitized mice. Figure S1 shows the sorting plot of dissociated lung cells. Figure S2 shows post-sort purity tests for sorted hrGFP^+^ and hrGFP^+^DsRed^+^ cells from the lungs of α*SMA-hrGFP+;NG2-DsRed+* mice. Figure S3 shows the examination of appropriate marker expression in sorted cell population. Figure S4 shows characterization of physical properties of BSMCs from a mouse model of chronic asthma. Figure S5 shows the concordance of our BSMCs array results with sets of genes identified as differentially expressed by previous asthma-related studies.
(PDF)
