# Supplementary material for: A New Approach for the Study of Lung Smooth Muscle Phenotypes and Its Application in a Murine Model of Allergic Airway Inflammation
Source: PLoS One. 2013 Sep 9;8(9):e74469. doi: 10.1371/journal.pone.0074469 (PMC3767675; doi:10.1371/journal.pone.0074469)
Supplement: File S1 — All supporting materials, including supplementary methods, tables, and figures are included in File S1. Table S1 lists all primer sequences for real-time PCR. Table S2 lists all deregulated gene list in BSMCs from OVA sensitized mice. Figure S1 shows the sorting plot of dissociated lung cells. Figure S2 shows post-sort purity tests for sorted hrGFP+ and hrGFP+DsRed+ cells from the lungs of αSMA-hrGFP+;NG2-DsRed+ mice. Figure S3 shows the examination of appropriate marker expression in sorted cell population. Figure S4 shows characterization of physical properties of BSMCs from a mouse model of chronic asthma. Figure S5 shows the concordance of our BSMCs array results with sets of genes identified as differentially expressed by previous asthma-related studies. (PDF) [file pone.0074469.s001.pdf]

Supporting Materials

Supplementary Methods

Induction of chronic asthma. Mice were sensitized by two IP injections of OVA (50 μg) on day 0 and day 7. Mice were then subjected to OVA challenge by nebulization (2% OVA in PBS) for 5 consecutive days between day 14 and day 18 followed by a 9-day rest. The same two-week treatment, 5-day nebulization followed by 9-day rest, was repeated 4 more times before mice were analyzed. Control mice received OVA sensitization but not challenge.

Supplementary Tables

Table S1. Primer sequences for real-time PCR.

Gene	Sybr/Taqman	Sequence / Reference #	Amplicon (bp)
**Cxcl5**	Sybr	5'AGCATCTAGCTGAAGCTGCCCC 3'<br>5'CCGTAGGGCACTGTGGACCTG 3'	141
**IL13Rα2**	Sybr	5'TGGGAAGGTTACACAGGGCCAG 3'<br>5'TGGAGGCTCAATGTGGGTTCAGGT 3'	131
**Lcn2**	Sybr	5'TCCGGGGCAGGTGGTACGTT 3'<br>5'CAGCCCTGGTCCTGGTCCCTGA 3'	154
**Cyp2e1**	Sybr	5'ACCTGCCCCCAGGACCTTTCC 3'<br>5'CCGAAGCGCTTTGCCAACTTGGTT 3'	103
**GapDH**	Sybr	5'AACCAGAAGACTGTGGATGG 3'<br>5'CACATTGGGGGTAGGAACAC 3'	171
**Col6α5**	Taqman	Hs00542046_m1	110
**Adrb1**	Taqman	Mm00431701_s1	76
**Adrb2**	Taqman	Mm02524224_s1	75

Table S2. Deregulated gene list in BSMCs from OVA sensitized mice.

The 443 genes with *p*-value < 0.01 for OVA vs. PBS control comparisons are listed, ordered by *p*-value. FDR is the associated false-discovery rate. LFC denotes $\log_2$ fold-change for OVA-sensitized arrays versus control arrays: negative LFC indicates down-regulation in BSMCs from OVA sensitized mice, and positive LFC indicates up-regulation. The first column gives the Affymetrix MoGene-1_0-st-v1.r4 probeset ID for the gene.

Probeset	SYMBOL	UNIGENE	ENTREZID	REFSEQ	LFC	p-value	FDR
10496001	Cfi	Mm.117180	12630	NM_007686	2.05	3.40E-06	0.071
10607206	Il13ra2	Mm.368330	16165	NM_008356	2.11	2.10E-05	0.22
10410007	Fbp1	Mm.423078	14121	NM_019395	2.22	3.20E-05	0.22
10496425	Adh7	Mm.8473	11529	NM_009626	-1.04	4.90E-05	0.23
10399854	Slc26a4	Mm.100187	23985	NM_011867	1.41	7.00E-05	0.23
10606355	Cysltr1	Mm.287166	58861	NM_021476	2.13	7.90E-05	0.23
10530421	Gabra4	Mm.248731	14397	NM_010251	-0.78	8.10E-05	0.23
10551293	Cyp2f2	Mm.4515	13107	NM_007817	-1.04	9.50E-05	0.23
10436100	Retnlg	Mm.231288	245195	NM_181596	1.01	1.00E-04	0.23
10498921	Tdo2	Mm.258622	56720	NM_019911	2.33	0.00011	0.23
10522324	Gabrb1	Mm.38567	14400	NM_008069	-0.96	0.00014	0.24
10492021	Postn	Mm.236067	50706	NM_001198765	0.67	0.00016	0.24
10400304	Egln3	Mm.133037	112407	NM_028133	1.88	0.00016	0.24
10596403	Col6a5	Mm.39826	665033	NM_001167923	3.5	0.00016	0.24
10438904	Lrrc15	Mm.290677	74488	NM_028973	1.17	0.00022	0.31
10599463	Xpnpep2	Mm.129279	170745	NM_133213	-0.82	0.00024	0.31
10565910	Plekhb1	Mm.26633	27276	NM_001163182	-0.71	0.00027	0.33
10502565	Clca2	Mm.20897	80797	NM_030601	1.37	0.00035	0.35
10379524	Ccl11	Mm.4686	20292	NM_011330	1.16	0.00036	0.35
10419568	Ear11	Mm.153688	93726	NM_053113	4.2	0.00037	0.35
10403413	Idi1	Mm.29847	319554	NM_145360	0.84	0.00037	0.35
10432492	Faim2	Mm.39760	72393	NM_001038658	-0.67	4.00E-04	0.35
10417212	Itgbl1	Mm.383176	223272	NM_145467	-0.97	0.00042	0.35
10462435	Mlana	Mm.138437	77836	NM_029993	-0.68	0.00042	0.35
10542335	Gprc5a	Mm.23575	232431	NM_181444	-0.66	0.00043	0.35
10495136	Chia	Mm.46418	81600	NM_023186	2.33	0.00044	0.35
10363415	Spock2	Mm.153429	94214	NM_052994	-1	0.00048	0.35
10402435	Serpina3c	Mm.422814	16625	NM_008458	1.08	5.00E-04	0.35
10471216	Lamc3	Mm.302362	23928	NM_011836	-0.67	5.00E-04	0.35
10451123	Slc29a1	Mm.29744	63959	NM_001199113	-0.56	0.00053	0.35
10403291	Akr1c14	Mm.26838	105387	NM_134072	-0.88	0.00058	0.35

10389929	Cacna1g	Mm.29585	12291	NM_001112813	-0.59	0.00059	0.35
10534493	Ccl24	Mm.31505	56221	NM_019577	1.45	6.00E-04	0.35
10470014	Entpd2	Mm.482100	12496	NM_009849	-0.69	6.00E-04	0.35
10352867	Plxna2	Mm.2251	18845	NM_008882	-0.57	0.00061	0.35
10494817	Ngf	Mm.1259	18049	NM_001112698	-0.76	0.00062	0.35
10511835	Fhl5	Mm.87325	57756	NM_021318	0.8	0.00065	0.35
10501622	Gpr88	Mm.40025	64378	NM_022427	0.61	0.00065	0.35
10523120	Cxcl5	Mm.4660	20311	NM_009141	1.99	0.00066	0.35
10446229	Tnfsf9	Mm.41171	21950	NM_009404	-0.78	0.00067	0.35
10471247	Aif1l	Mm.24838	108897	NM_145144	1.39	0.00069	0.35
10441233	Mx1	Mm.33996	17857	NM_010846	0.79	7.00E-04	0.35
10502816	Gipc2	Mm.97	54120	NM_016867	-0.52	0.00073	0.35
10564417	Aldh1a3	Mm.140988	56847	NM_053080	1.65	0.00078	0.37
10459229	Pcyox1l	Mm.234457	240334	NM_172832	1.07	0.00082	0.38
10421810	1190002H23Rik	Mm.29811	66214	NM_025427	-0.56	0.00084	0.38
10559580	Syt5	Mm.358663	53420	NM_016908	-0.67	0.00085	0.38
10386211	Fam183b	Mm.296819	75429	NM_001162878	-0.89	0.00087	0.38
10497066	Zranb2	Mm.259036	53861	NM_017381	0.6	0.00091	0.38
10599335	Mcts1	Mm.262453	68995	NM_026902	0.58	0.00095	0.38
10502805	Ptgfr	Mm.331442	19220	NM_008966	-0.82	0.00096	0.38
10479938	Echdc3	Mm.38342	67856	NM_024208	0.9	0.00096	0.38
10496656	Col24a1	Mm.235570	71355	NM_027770	0.56	0.001	0.4
10525932	Tmem132c	Mm.444235	208213	NM_175432	-0.76	0.001	0.4
10401109	Gpx2	Mm.441856	14776	NM_030677	-0.63	0.0011	0.4
10396952	Ttc9	Mm.130002	69480	NM_001033149	-0.83	0.0011	0.4
10403073	Ighg	Mm.342177	380794	XM_001472541	1.34	0.0011	0.4
10538187	Gpnmb	Mm.302602	93695	NM_053110	-1.09	0.0011	0.4
10469255	Prkcq	Mm.329993	18761	NM_008859	0.94	0.0012	0.4
10491721	Spry1	Mm.330986	24063	NM_011896	-0.48	0.0012	0.4
10393904	Notum	Mm.32839	77583	NM_175263	-0.75	0.0012	0.4
10391444	Rundc1	Mm.490557	217201	NM_172566	0.77	0.0013	0.4
10564624	St8sia2	Mm.4954	20450	NM_009181	-0.85	0.0013	0.4
10413813	Galntl2	Mm.40681	78754	NM_030166	-1	0.0013	0.4
10356886	Slco4c1	Mm.11662	227394	NM_172658	0.61	0.0014	0.4
10498653	1110032A04Rik	Mm.45481	66183	NM_001164210	-0.51	0.0014	0.4
10427391	Ewsr1	Mm.142822	14030	NM_007968	0.47	0.0014	0.4
10365344	Tcp11l2	Mm.296142	216198	NM_146008	-0.45	0.0014	0.4
10487011	Gatm	Mm.29975	67092	NM_025961	1.8	0.0014	0.4
10351455	Rgs5	Mm.20954	19737	NM_009063	1.15	0.0014	0.4
10486255	Oip5	Mm.246412	70645	NM_001042653	0.49	0.0015	0.4
10569429	Cdkn1c	Mm.168789	12577	NM_001161624	-0.49	0.0016	0.4
10526783	Nxpe5	Mm.156846	381680	NM_001013773	0.44	0.0016	0.4
10351873	Pyhin1	Mm.447	236312	NM_175026	1.19	0.0016	0.4

10432918	Krt8	Mm.358618	16691	NM_031170	-0.83	0.0016	0.4
10460468	Ctsf	Mm.29561	56464	NM_019861	-0.55	0.0016	0.4
10365891	Tmcc3	Mm.23047	319880	NM_001168684	-0.5	0.0017	0.4
10533050	Hspb8	Mm.21549	80888	NM_030704	-0.43	0.0017	0.4
10551282	Cyp2a5	Mm.389848	13087	NM_007812	-0.49	0.0017	0.4
10376868	Trpv2	Mm.288064	22368	NM_011706	0.98	0.0017	0.4
10502359	Dapp1	Mm.254835	26377	NM_011932	0.88	0.0017	0.4
10458323	Nrg2	Mm.380390	100042150	NM_001167891	-0.6	0.0018	0.4
10608681	Gm4841	Mm.329460	225594	NM_001034859	0.83	0.0018	0.4
10420668	Mir15a	NA	387174	NR_029733	-0.61	0.0018	0.4
10518526	Angptl7	Mm.388929	654812	NM_001039554	-1.29	0.0018	0.4
10583314	Taf1d	Mm.354115	75316	NM_026541	0.46	0.0018	0.4
10376326	Irgm2	Mm.458517	54396	NM_019440	0.46	0.0019	0.4
10451641	9830107B12Rik	Mm.466999	328829	NM_001177896	0.49	0.0019	0.4
10519998	Lrrc17	Mm.439963	74511	NM_028977	-0.59	0.0019	0.4
10420483	Phf11	Mm.254918	219131	NM_172603	0.53	0.002	0.4
10362186	Moxd1	Mm.285934	59012	NM_021509	1.71	0.002	0.4
10414271	Ptger2	Mm.4630	19217	NM_008964	0.62	0.002	0.4
10467206	Ppp1r3c	Mm.24724	53412	NM_016854	0.56	0.002	0.4
10412729	Sntn	Mm.39919	218739	NM_177624	-1.15	0.002	0.4
10555510	Pde2a	Mm.247564	207728	NM_001008548	0.91	0.002	0.4
10475517	AA467197	Mm.36783	433470	NM_001004174	1.27	0.002	0.4
10462973	Hells	Mm.392920	15201	NM_008234	0.7	0.002	0.4
10575873	Osgin1	Mm.272357	71839	NM_027950	-0.61	0.0021	0.4
10398039	Serpina3f	Mm.483584	238393	NM_001033335	1.73	0.0021	0.4
10501026	Chi3l4	Mm.425140	104183	NM_145126	1.28	0.0021	0.4
10493640	Nup210l	Mm.159949	77595	NM_029937	-0.64	0.0021	0.4
10598093	Tarm1	Mm.245747	245126	NM_177363	0.74	0.0022	0.4
10395356	Agr3	Mm.190508	403205	NM_207531	-0.95	0.0022	0.4
10498018	Pcdh18	Mm.87246	73173	NM_130448	-0.5	0.0022	0.4
10356335	Nmur1	Mm.389159	14767	NM_010341	0.55	0.0023	0.4
10543524	Gcc1	Mm.332950	74375	NM_028900	-0.56	0.0023	0.4
10538791	Tnip3	Mm.117558	414084	NM_001001495	1.03	0.0023	0.4
10462922	Plce1	Mm.34031	74055	NM_019588	-0.77	0.0023	0.4
10540298	Chl1	Mm.251288	12661	NM_007697	2.08	0.0023	0.4
10453055	Gm10494	Mm.414347	100038718	NR_033462	0.55	0.0023	0.4
10570291	F10	Mm.262589	14058	NM_001242368	0.71	0.0023	0.4
10385518	Tgtp1	Mm.15793	21822	NM_011579	0.46	0.0023	0.4
10448117	Has1	Mm.255701	15116	NM_008215	-0.9	0.0023	0.4
10407281	Esm1	Mm.38929	71690	NM_023612	-0.46	0.0024	0.4
10438769	Cldn1	Mm.289441	12737	NM_016674	-0.58	0.0024	0.4
10408162	Zfp322a	Mm.286454	218100	NM_001111107	0.44	0.0024	0.4
10408081	Hist1h1b	Mm.221314	56702	NM_020034	0.93	0.0024	0.4

10427026	Grasp	Mm.276573	56149	NM_019518	-0.52	0.0024	0.4
10366653	Wif1	Mm.32831	24117	NM_011915	-0.67	0.0024	0.4
10374083	Aebp1	Mm.4665	11568	NM_009636	-0.5	0.0024	0.4
10497051	Negr1	Mm.317293	320840	NM_001039094	-0.76	0.0025	0.4
10537785	Tas2r143	Mm.377921	387514	NM_001001452	-1.07	0.0025	0.4
10540359	Cntn4	Mm.321683	269784	NM_001109749	-0.62	0.0025	0.4
10447702	Ppih	Mm.304080	66101	NM_001110129	0.46	0.0025	0.4
10444890	Ier3	Mm.25613	15937	NM_133662	-0.53	0.0025	0.4
10541587	Clec4a2	Mm.47384	26888	NM_001170332	0.6	0.0025	0.4
10510047	Gm10565	Mm.402714	100038693	NR_027137	0.42	0.0026	0.4
10367600	Esr1	Mm.463262	13982	NM_007956	-0.46	0.0026	0.4
10492558	Smc4	Mm.206841	70099	NM_133786	0.51	0.0026	0.4
10491477	Sox2	Mm.65396	20674	NM_011443	-0.58	0.0026	0.4
10537787	Tas2r135	Mm.377919	387512	NM_199159	-1.22	0.0026	0.4
10359948	Uap1	Mm.27969	107652	NM_133806	-0.59	0.0026	0.4
10392739	Sdk2	Mm.130504	237979	NM_172800	-0.65	0.0026	0.4
10542156	Clec2d	Mm.197536	93694	NM_053109	0.53	0.0027	0.4
10509568	Camk2n1	Mm.41603	66259	NM_025451	-0.73	0.0027	0.4
10408870	Tbc1d7	Mm.269213	67046	NM_001252639	0.85	0.0027	0.4
10436783	Sod1	Mm.276325	20655	NM_011434	-0.4	0.0027	0.4
10478525	Wfdc2	Mm.27289	67701	NM_026323	-0.56	0.0027	0.4
10451805	Sgol1	Mm.153202	72415	NM_028232	0.62	0.0027	0.4
10551226	Cyp2a4	Mm.154643	13086	NM_009997	-0.73	0.0028	0.4
10404063	Hist1h2ab	Mm.423402	319172	NM_175660	1.55	0.0028	0.4
10578916	Sc4mol	Mm.30119	66234	NM_025436	0.59	0.0028	0.4
10393449	Socs3	Mm.3468	12702	NM_007707	-0.51	0.0028	0.4
10538459	Aqp1	Mm.18625	11826	NM_007472	-0.6	0.0028	0.4
10434089	Ccdc74a	Mm.297777	72315	NM_001166164	-0.45	0.0028	0.4
10494632	Adam30	Mm.50711	71078	NM_027665	-0.42	0.0029	0.4
10570483	Arhgef10	Mm.240298	234094	NM_001037736	-0.42	0.0029	0.4
10363231	Smpdl3a	Mm.2379	57319	NM_020561	-0.45	0.0029	0.4
10541071	8430408G22Rik	Mm.38231	213393	NM_001166580	-0.73	0.0029	0.4
10524327	Mn1	Mm.332576	433938	NM_001081235	-0.41	0.0029	0.41
10607225	Lrch2	Mm.30506	210297	NM_001081173	0.76	0.003	0.41
10364035	Gm5779	Mm.349862	544707	NR_033602	0.43	0.003	0.41
10435043	Tm4sf19	Mm.484240	277203	NM_001160402	0.55	0.003	0.41
10531994	Gbp6	Mm.275893	100702	NM_194336	0.53	0.003	0.41
10489701	Ocstamp	Mm.72179	74614	NM_029021	0.46	0.003	0.41
10513739	Tnc	Mm.454219	21923	NM_011607	1.04	0.0031	0.41
10544417	Epha1	Mm.133330	13835	NM_023580	-0.52	0.0031	0.41
10500938	Wnt2b	Mm.10740	22414	NM_009520	-0.55	0.0032	0.41
10463476	Kazald1	Mm.24417	107250	NM_178929	-0.43	0.0032	0.41
10399087	Ncapg2	Mm.21516	76044	NM_133762	0.51	0.0033	0.41

10467319	Rbp4	Mm.2605	19662	NM_001159487	-0.47	0.0033	0.41
10525158	Oas1b	Mm.233471	23961	NM_001083925	0.69	0.0033	0.41
10416251	Egr3	Mm.103737	13655	NM_018781	-0.62	0.0033	0.41
10572461	Gm16486	Mm.462671	637079	XM_912668	-0.58	0.0033	0.41
10453738	Fzd8	Mm.184289	14370	NM_008058	-0.46	0.0033	0.41
10349157	Serpinb2	Mm.271870	18788	NM_001174170	0.89	0.0033	0.41
10352000	Kmo	Mm.27217	98256	NM_133809	0.93	0.0034	0.42
10585286	Arhgap20	Mm.400352	244867	NM_175535	-0.69	0.0034	0.42
10405633	Ntrk2	Mm.130054	18212	NM_001025074	-0.9	0.0034	0.42
10406536	Tmem167	Mm.241387	66074	NM_025335	0.46	0.0034	0.42
10493850	Sprr2a1	Mm.6853	20755	NM_011468	0.4	0.0035	0.42
10404069	Hist1h1a	Mm.377137	80838	NM_030609	0.58	0.0035	0.42
10608655	H60a	Mm.387042	15101	NM_010400	0.56	0.0036	0.42
10438753	Leprel1	Mm.483044	210530	NM_173379	0.86	0.0036	0.42
10563108	Snord35a	NA	27211	NR_000003	0.68	0.0036	0.42
10375838	Col23a1	Mm.154093	237759	NM_153393	-0.41	0.0036	0.42
10542959	Bet1	Mm.286457	12068	NM_009748	0.4	0.0036	0.42
10450161	H2-Ea-ps	Mm.15680	100504404	NM_010381	0.6	0.0036	0.42
10361089	Fam71a	Mm.61148	619288	NM_001109759	-0.9	0.0036	0.42
10429491	Arc	Mm.25405	11838	NM_018790	-0.54	0.0036	0.42
10416023	Scara5	Mm.83840	71145	NM_001168318	-0.91	0.0036	0.42
10421877	Diap3	Mm.440585	56419	NM_019670	0.83	0.0037	0.42
10441718	Park2	Mm.311110	50873	NM_016694	-0.43	0.0037	0.42
10517600	Pink1	Mm.18539	68943	NM_026880	-0.39	0.0037	0.42
10606333	Fndc3c1	Mm.298000	333564	NM_001007580	0.76	0.0037	0.42
10549108	Abcc9	Mm.35670	20928	NM_001044720	-0.45	0.0038	0.42
10404874	Mylip	Mm.212855	218203	NM_153789	-0.52	0.0038	0.42
10499130	Rnu73b	NA	19871	NR_004418	0.61	0.0038	0.42
10482172	Zbtb26	Mm.383299	320633	NM_199025	0.39	0.0039	0.42
10558496	Lrrc27	Mm.17757	76612	NM_001143755	0.79	0.0039	0.42
10428672	Dscc1	Mm.486550	72107	NM_183089	0.4	0.0039	0.42
10377774	Mgl2	Mm.222465	216864	NM_145137	1.07	0.004	0.42
10368041	3110003A17Rik	Mm.276041	73112	NM_028440	0.45	0.004	0.42
10419167	Ptgdr	Mm.5105	19214	NM_008962	0.72	0.004	0.42
10599192	Lonrf3	Mm.327654	74365	NM_028894	-0.67	0.0041	0.42
10388430	Serpinf1	Mm.2044	20317	NM_011340	-0.41	0.0041	0.42
10354141	Lonrf2	Mm.487137	381338	NM_001029878	-0.49	0.0041	0.42
10497505	Nlgn1	Mm.316080	192167	NM_001163387	-0.4	0.0041	0.42
10595787	Atr	Mm.212462	245000	NM_019864	-0.38	0.0041	0.42
10544837	Tril	Mm.422727	66873	NM_025817	-0.63	0.0041	0.42
10608710	Ahsp	Mm.423023	170812	NM_133245	0.8	0.0041	0.42
10405918	Rsl1	Mm.284988	380855	NM_001013769	0.57	0.0042	0.42
10568536	Cpxm2	Mm.471654	55987	NM_018867	-0.42	0.0042	0.42

10388042	6330403K07Rik	Mm.27768	103712	NM_134022	-0.58	0.0042	0.42
10552743	Il4i1	Mm.2565	14204	NM_010215	0.82	0.0043	0.42
10426081	Fam19a5	Mm.244319	106014	NM_001252310	-0.71	0.0043	0.42
10416406	Htr2a	Mm.214351	15558	NM_172812	0.7	0.0043	0.42
10422537	Nalcn	Mm.255348	338370	NM_177393	-0.52	0.0043	0.42
10603087	Pir	Mm.293463	69656	NM_027153	-0.58	0.0043	0.42
10479902	Dhtkd1	Mm.222517	209692	NM_001081131	-0.38	0.0043	0.42
10479761	Meig1	Mm.2688	104362	NM_008579	-0.6	0.0043	0.42
10462398	Pdcd1lg2	Mm.116737	58205	NM_021396	1.02	0.0043	0.42
10531100	Sult1d1	Mm.6824	53315	NM_016771	-0.84	0.0043	0.42
10519913	Magi2	Mm.332231	50791	NM_001170745	-0.4	0.0043	0.42
10473058	Osbpl6	Mm.240435	99031	NM_145525	-0.6	0.0044	0.42
10421269	Sorbs3	Mm.5068	20410	NM_011366	-0.38	0.0044	0.42
10401607	Pgf	Mm.4809	18654	NM_008827	0.87	0.0044	0.42
10356601	Per2	Mm.482463	18627	NM_011066	-0.51	0.0044	0.42
10366043	Dusp6	Mm.1791	67603	NM_026268	-0.53	0.0044	0.42
10416958	Mir92-1	NA	751549	NR_029816	-0.49	0.0044	0.42
10398052	Serpina3h	Mm.390522	546546	NM_001034870	1.18	0.0045	0.42
10523145	Cxcl15	Mm.64326	20309	NM_011339	0.89	0.0045	0.42
10427772	Tars	Mm.286061	110960	NM_033074	0.45	0.0046	0.42
10374356	Vstm2a	Mm.284015	211739	NM_145967	-0.5	0.0046	0.42
10349529	Thsd7b	Mm.380518	210417	NM_172485	-0.38	0.0046	0.42
10476728	Dtd1	Mm.28109	66044	NM_025314	-0.38	0.0046	0.42
10531980	Gbp9	Mm.425261	236573	NM_172777	0.41	0.0047	0.43
10345025	Iars	Mm.21118	105148	NM_172015	0.42	0.0047	0.43
10379535	Ccl8	Mm.42029	20307	NM_021443	2.16	0.0048	0.43
10494114	Selenbp1	Mm.196558	20341	NM_009150	-0.68	0.0049	0.44
10582427	Cbfa2t3	Mm.194339	12398	NM_001109873	-0.65	0.0049	0.44
10533145	Tpcn1	Mm.114054	252972	NM_145853	-0.43	0.005	0.44
10424113	Mal2	Mm.410994	105853	NM_178920	-0.68	0.005	0.44
10551197	Cyp2b10	Mm.218749	13088	NM_009999	-0.96	0.005	0.44
10362073	Sgk1	Mm.28405	20393	NM_001161845	-0.45	0.005	0.44
10521824	Sod3	Mm.2407	20657	NM_011435	0.4	0.005	0.44
10408638	1700026J04Rik	Mm.380836	71864	NM_027967	-0.38	0.005	0.44
10497381	Cyp7b1	Mm.316000	13123	NM_007825	0.54	0.005	0.44
10355259	Myl1	Mm.1000	17901	NM_001113387	0.46	0.0051	0.44
10467897	Cyp2c44	Mm.329866	226143	NM_001001446	-0.55	0.0051	0.44
10427125	Igfbp6	Mm.358609	16012	NM_008344	-0.69	0.0051	0.44
10403943	Hist1h2bm	Mm.277425	319186	NM_178200	0.77	0.0051	0.44
10567355	Gprc5b	Mm.103439	64297	NM_001195774	-0.47	0.0051	0.44
10500011	Tuft1	Mm.10214	22156	NM_011656	-0.4	0.0051	0.44
10497203	Hey1	Mm.29581	15213	NM_010423	-0.41	0.0052	0.44
10550956	Ethe1	Mm.29553	66071	NM_023154	0.63	0.0052	0.44

10576495	Trim67	Mm.422769	330863	NM_198632	-0.41	0.0052	0.44
10354389	Slc39a10	Mm.233889	227059	NM_172653	-0.37	0.0053	0.44
10461454	1810006K21Rik	Mm.375100	69038	NM_001163431	0.4	0.0053	0.44
10414262	Ear2	Mm.327386	13587	NM_007895	1.27	0.0053	0.44
10530145	Tlr1	Mm.273024	21897	NM_030682	0.69	0.0053	0.44
10584208	Cdon	Mm.80509	57810	NM_021339	-0.47	0.0054	0.44
10428604	Tnfrsf11b	Mm.15383	18383	NM_008764	0.54	0.0054	0.44
10471555	Angptl2	Mm.208919	26360	NM_011923	-0.41	0.0054	0.44
10406968	Cenpk	Mm.281498	60411	NM_021790	0.76	0.0054	0.44
10435752	Lsamp	Mm.310524	268890	NM_175548	-0.47	0.0054	0.44
10412036	Apoo-ps	Mm.343319	621156	NR_004438	0.51	0.0054	0.44
10541885	Scnn1a	Mm.144114	20276	NM_011324	-0.64	0.0055	0.44
10498620	Trim59	Mm.490341	66949	NM_025863	0.41	0.0055	0.44
10368343	Arg1	Mm.154144	11846	NM_007482	0.83	0.0055	0.44
10371296	Glt8d2	Mm.191281	74782	NM_029102	-0.48	0.0055	0.44
10439114	Iqcg	Mm.485493	69707	NM_178378	-0.75	0.0055	0.44
10571680	Mlf1ip	Mm.217385	71876	NM_027973	0.55	0.0056	0.44
10508382	Ak2	Mm.29460	11637	NM_001033966	0.58	0.0056	0.44
10425283	Maff	Mm.86646	17133	NM_010755	-0.78	0.0056	0.44
10586865	Aldh1a2	Mm.42016	19378	NM_009022	0.77	0.0056	0.44
10458052	Epb4.1l4a	Mm.3465	13824	NM_013512	-0.48	0.0057	0.45
10504424	Reck	Mm.331573	53614	NM_016678	-0.55	0.0058	0.45
10505489	Pappa	Mm.317854	18491	NM_021362	-0.54	0.0058	0.45
10432972	Rarg	Mm.1273	19411	NM_001042727	-0.34	0.0059	0.45
10561128	Cyp2s1	Mm.275188	74134	NM_028775	-1.33	0.0059	0.45
10605431	Rab39b	Mm.45148	67790	NM_175122	1.29	0.0059	0.45
10504957	Smc2	Mm.2999	14211	NM_008017	0.55	0.0059	0.45
10554969	Odz4	Mm.254610	23966	NM_011858	-0.41	0.006	0.45
10438517	Alg3	Mm.24950	208624	NM_145939	0.69	0.006	0.45
10529875	Ldb2	Mm.25785	16826	NM_001077398	-0.34	0.006	0.45
10447294	Prkce	Mm.24614	18754	NM_011104	-0.41	0.006	0.45
10462281	Vldlr	Mm.4141	22359	NM_001161420	-0.66	0.006	0.45
10388254	Aspa	Mm.293574	11484	NM_023113	-0.74	0.006	0.45
10532019	Gbp11	Mm.458435	634650	NM_001039647	0.39	0.0061	0.45
10378068	Xaf1	Mm.291131	327959	NM_001037713	0.49	0.0061	0.45
10576807	Cd209d	Mm.111026	170779	NM_130904	0.94	0.0061	0.45
10546853	Srgap3	Mm.236401	259302	NM_080448	-0.4	0.0061	0.45
10576010	Gse1	Mm.334856	382034	NM_001145896	-0.38	0.0061	0.45
10479925	5430407P10Rik	Mm.133542	227545	NM_001159657	0.65	0.0062	0.45
10466728	1700028P14Rik	Mm.443287	67483	NM_026188	-0.67	0.0062	0.45
10466530	Pcsk5	Mm.3401	18552	NM_001163144	0.38	0.0063	0.45
10533345	Aldh2	Mm.284446	11669	NM_009656	-0.36	0.0063	0.45
10372226	Myf5	Mm.4984	17877	NM_008656	-0.5	0.0063	0.45

10344966	Ly96	Mm.116844	17087	NM_001159711	0.49	0.0063	0.45
10541564	Clec4a3	Mm.25684	73149	NM_001204241	1.17	0.0063	0.45
10458569	Nr3c1	Mm.129481	14815	NM_008173	-0.4	0.0063	0.45
10540401	Lrrn1	Mm.428543	16979	NM_008516	-0.66	0.0063	0.45
10523563	Cds1	Mm.46764	74596	NM_173370	-0.5	0.0064	0.45
10422436	Dock9	Mm.441054	105445	NM_001081039	-0.54	0.0064	0.45
10483679	Gpr155	Mm.465902	68526	NM_001080707	-0.52	0.0064	0.45
10445338	Enpp5	Mm.30145	83965	NM_001168620	-0.43	0.0065	0.45
10478258	Lpin3	Mm.292111	64899	NM_001199118	0.43	0.0065	0.45
10457475	Abhd3	Mm.273108	106861	NM_134130	-0.4	0.0066	0.46
10348632	Twist2	Mm.9474	13345	NM_007855	-0.51	0.0066	0.46
10368409	Lama2	Mm.256087	16773	NM_008481	-0.7	0.0067	0.46
10404061	Hist1h2bb	Mm.390567	319178	NM_175664	0.92	0.0067	0.46
10429506	Lypd2	Mm.306880	68311	NM_026671	-0.37	0.0067	0.46
10487340	Ncaph	Mm.29786	215387	NM_144818	0.9	0.0067	0.46
10432032	Vdr	Mm.245084	22337	NM_009504	0.74	0.0067	0.46
10476945	Cst7	Mm.12965	13011	NM_009977	0.58	0.0068	0.46
10593634	Elmod1	Mm.259791	270162	NM_177769	-0.83	0.0068	0.46
10405892	Gm10767	Mm.405005	100038538	NM_001177750	0.51	0.0068	0.46
10496251	Bdh2	Mm.45121	69772	NM_001172055	-0.46	0.0069	0.46
10394971	Klf11	Mm.9616	194655	NM_178357	-0.34	0.0069	0.46
10581289	Atp6v0d1	Mm.17708	11972	NM_013477	0.59	0.007	0.46
10495574	Sass6	Mm.440836	72776	NM_028349	0.51	0.007	0.46
10492689	Pdgfc	Mm.331089	54635	NM_019971	0.55	0.007	0.46
10438415	Iglv1	Mm.326349	16142	NA	0.82	0.007	0.46
10535053	Prkar1b	Mm.306163	19085	NM_001253890	-0.34	0.007	0.46
10471814	Gpr21	Mm.445325	338346	NM_177383	0.5	0.007	0.46
10444957	Ppp1r10	Mm.29385	52040	NM_001163818	-0.42	0.007	0.46
10436947	Kcne2	Mm.156736	246133	NM_134110	-0.34	0.007	0.46
10495625	Dpyd	Mm.27907	99586	NM_170778	-0.66	0.007	0.46
10521731	Ncapg	Mm.32012	54392	NM_019438	0.76	0.0071	0.46
10358978	Ier5	Mm.12246	15939	NM_010500	-0.46	0.0071	0.46
10588226	Amotl2	Mm.21145	56332	NM_019764	-0.39	0.0071	0.46
10548375	Clec7a	Mm.239516	56644	NM_020008	1.18	0.0072	0.46
10608675	Serpina3k	Mm.291569	20714	NM_011458	0.89	0.0072	0.46
10378749	1700016K19Rik	Mm.271247	74230	NM_198637	-0.6	0.0073	0.46
10603266	Nudt10	Mm.426101	102954	NM_001031664	-0.45	0.0073	0.46
10602033	Cldn2	Mm.117068	12738	NM_016675	0.96	0.0073	0.46
10580282	Junb	Mm.1167	16477	NM_008416	-0.38	0.0073	0.46
10346191	Stat1	Mm.277406	20846	NM_001205313	0.4	0.0074	0.46
10606366	Zcchc5	Mm.85581	213436	NM_199468	-0.64	0.0074	0.46
10418247	Pde12	Mm.290758	211948	NM_178668	-0.66	0.0075	0.46
10469110	Usp6nl	Mm.40673	98910	NM_001080548	0.38	0.0075	0.46

10433885	Cebpd	Mm.347407	12609	NM_007679	-0.6	0.0075	0.46
10527936	Fzd1	Mm.246003	14362	NM_021457	-0.33	0.0075	0.46
10425299	Tomm22	Mm.246435	223696	NM_172609	0.42	0.0075	0.46
10566350	Trim30b	Mm.347647	244183	NM_175648	0.41	0.0076	0.46
10428066	1700084J12Rik	Mm.483393	73486	NM_028551	0.36	0.0076	0.46
10381776	Mapt	Mm.1287	17762	NM_001038609	-0.43	0.0076	0.46
10509267	Wnt4	Mm.20355	22417	NM_009523	-0.34	0.0076	0.46
10569504	Tnfrsf23	Mm.290780	79201	NM_024290	0.87	0.0076	0.46
10534142	Sbds	Mm.280484	66711	NM_023248	-0.36	0.0077	0.46
10537789	Tas2r126	Mm.377914	387353	NM_207028	-0.53	0.0077	0.46
10530059	Sel1l3	Mm.235020	231238	NM_172710	1.16	0.0077	0.46
10538163	Abp1	Mm.213898	76507	NM_001161621	1.46	0.0077	0.46
10549647	Ncr1	Mm.240231	17086	NM_010746	0.56	0.0077	0.46
10497582	Tmem212	Mm.59189	208613	NM_001164437	-0.78	0.0077	0.46
10346365	Sgol2	Mm.32800	68549	NM_001177867	0.76	0.0078	0.46
10504234	Unc13b	Mm.128892	22249	NM_001081413	-0.37	0.0078	0.46
10458663	Dpysl3	Mm.428551	22240	NM_001136086	0.42	0.0079	0.46
10494085	Selenbp2	Mm.225405	20342	NM_019414	-0.59	0.0079	0.46
10508734	Ptafr	Mm.89389	19204	NM_001081211	0.83	0.0079	0.46
10426110	Pim3	Mm.400129	223775	NM_145478	-0.46	0.0079	0.46
10469816	Il1rn	Mm.882	16181	NM_001039701	0.8	0.008	0.46
10587315	Gsta4	Mm.2662	14860	NM_010357	-0.59	0.008	0.46
10598976	Timp1	Mm.8245	21857	NM_001044384	1.14	0.008	0.46
10535006	BC037034	Mm.164493	231807	NM_153161	0.49	0.008	0.46
10400546	Fbxo33	Mm.311026	70611	NM_001033156	-0.35	0.008	0.46
10583145	Tmem123	Mm.283293	71929	NM_133739	0.38	0.008	0.46
10491962	Foxo1	Mm.29891	56458	NM_019739	-0.37	0.008	0.46
10491860	Phf17	Mm.286285	269424	NM_001130184	-0.4	0.008	0.46
10383088	Gaa	Mm.4793	14387	NM_001159324	-0.39	0.008	0.46
10608698	Zfp429	Mm.303790	72807	NM_001080941	0.4	0.0081	0.46
10556141	Olfr495	Mm.223052	258361	NM_146364	-0.37	0.0082	0.46
10419156	Ear10	Mm.429087	93725	NM_053112	1.18	0.0082	0.46
10353252	Gm106	Mm.131665	226866	NM_001033288	1.09	0.0082	0.46
10565067	Nmb	Mm.22246	68039	NM_026523	-0.37	0.0082	0.46
10583519	Icam1	Mm.435508	15894	NM_010493	-0.43	0.0084	0.46
10525591	Kntc1	Mm.441089	208628	NM_001042421	0.8	0.0084	0.46
10436095	Retnla	Mm.441868	57262	NM_020509	2.7	0.0085	0.46
10362472	Rsph4a	Mm.131949	212892	NM_001162957	-1.1	0.0085	0.46
10604832	Mir505	NA	751545	NR_030499	-0.91	0.0085	0.46
10409061	Mirlet7f-1	NA	387252	NR_029731	-0.34	0.0085	0.46
10428302	Klf10	Mm.4292	21847	NM_013692	-0.58	0.0085	0.46
10477970	Src	Mm.22845	20779	NM_001025395	0.37	0.0085	0.46
10580183	Ier2	Mm.399	15936	NM_010499	-0.51	0.0085	0.46

10502898	Ube2l3	Mm.3074	22195	NM_009456	0.35	0.0086	0.46
10398069	Serpina3m	Mm.395085	20717	NM_009253	1.07	0.0086	0.46
10435457	Parp9	Mm.49074	80285	NM_030253	0.35	0.0086	0.46
10518847	Phf13	Mm.25582	230936	NM_172705	-0.34	0.0086	0.46
10585331	Exph5	Mm.277540	320051	NM_176846	-0.44	0.0087	0.46
10397332	Eif2b2	Mm.29041	217715	NM_145445	0.48	0.0087	0.46
10448803	Hn1l	Mm.371601	52009	NM_198937	0.73	0.0087	0.46
10475866	Bcl2l11	Mm.141083	12125	NM_009754	-0.37	0.0088	0.46
10408077	Hist1h2ak	Mm.370338	319169	NM_178183	1.04	0.0088	0.46
10371400	Cry1	Mm.26237	12952	NM_007771	-0.48	0.0088	0.46
10582180	Fam92b	Mm.334852	436062	NM_001033980	-0.48	0.0088	0.46
10569008	Cox8b	Mm.3841	12869	NM_007751	-0.58	0.0088	0.46
10385248	Hmmr	Mm.116997	15366	NM_013552	1.03	0.0088	0.46
10493626	1700094D03Rik	Mm.482512	73545	NM_028567	-0.58	0.0088	0.46
10490150	Zbp1	Mm.116687	58203	NM_001139519	0.76	0.0088	0.46
10533246	Oas1g	Mm.389688	23960	NM_011852	0.47	0.0088	0.46
10542028	Fgf23	Mm.347933	64654	NM_022657	1.41	0.0088	0.46
10403948	Hist1h2be	Mm.440391	319179	NM_001177653	0.71	0.0088	0.46
10419154	Ear1	Mm.86948	13586	NM_007894	1.07	0.0088	0.46
10510482	Clstn1	Mm.38993	65945	NM_023051	-0.38	0.0088	0.46
10422227	Spry2	Mm.489870	24064	NM_011897	-0.47	0.0088	0.46
10503695	Bach2	Mm.431426	12014	NM_001109661	-0.37	0.0089	0.46
10538408	2410066E13Rik	Mm.233860	68235	NM_026629	-0.55	0.0089	0.47
10533751	Pitpnm2	Mm.44261	19679	NM_011256	-0.37	0.009	0.47
10472300	Psmd14	Mm.218198	59029	NM_021526	0.36	0.009	0.47
10363735	Egr2	Mm.290421	13654	NM_010118	-0.42	0.009	0.47
10387155	Wdr16	Mm.441823	71860	NM_027963	-0.64	0.009	0.47
10477450	Bpifa2	Mm.29197	19194	NM_008953	-0.79	0.0091	0.47
10403978	Hist1h2br	Mm.471247	665622	NM_001110555	0.74	0.0091	0.47
10586118	Calml4	Mm.440576	75600	NM_001102468	-0.46	0.0093	0.47
10370931	Mknk2	Mm.42126	17347	NM_021462	-0.33	0.0093	0.47
10590888	Gm7520	NA	665154	XR_031035	0.35	0.0093	0.47
10473160	Ssfa2	Mm.272881	70599	NM_080558	-0.36	0.0093	0.47
10417544	Acox2	Mm.28700	93732	NM_001161667	-0.46	0.0093	0.47
10500999	1700027A23Rik	Mm.386825	100503311	NM_001200028	-0.4	0.0094	0.47
10466210	Ms4a6d	Mm.290390	68774	NM_026835	1.23	0.0094	0.47
10512254	1110017D15Rik	Mm.45614	73721	NM_001048005	-0.64	0.0094	0.47
10513008	Klf4	Mm.4325	16600	NM_010637	-0.41	0.0095	0.47
10408085	Hist1h2an	Mm.377877	319170	NM_178184	0.53	0.0095	0.47
10501555	Amy1	Mm.439727	11722	NM_001110505	-0.68	0.0095	0.47
10591978	Ntm	Mm.283138	235106	NM_172290	0.44	0.0096	0.47
10558673	Cyp2e1	Mm.21758	13106	NM_021282	-0.67	0.0096	0.47
10550698	Ckm	Mm.2375	12715	NM_007710	0.39	0.0096	0.47

10595753	Trpc1	Mm.149633	22063	NM_011643	-0.43	0.0096	0.47
10433492	Atf7ip2	Mm.108729	75329	NM_029253	-0.31	0.0096	0.47
10407159	Ankrd55	Mm.159895	77318	NM_001168403	0.43	0.0096	0.47
10426894	Mettl7a3	Mm.425943	668178	NM_001081471	-0.46	0.0097	0.47
10397166	Acot5	Mm.484019	217698	NM_145444	-0.35	0.0097	0.47
10466947	Ermp1	Mm.267131	226090	NM_001081213	-0.43	0.0097	0.47
10452709	Ndc80	Mm.225956	67052	NM_023294	0.57	0.0098	0.47
10447732	Pacrg	Mm.18889	69310	NM_027032	-0.34	0.0098	0.47
10542953	Tfpi2	Mm.25612	21789	NM_009364	1.06	0.0098	0.47
10428918	9930014A18Rik	Mm.230853	320469	NM_177166	-0.37	0.0098	0.47
10587880	Pcolce2	Mm.46016	76477	NM_029620	-0.57	0.0098	0.47
10488378	Thbd	Mm.24096	21824	NM_009378	-0.62	0.0098	0.47
10385583	Ltc4s	Mm.245151	17001	NM_008521	0.53	0.0098	0.47
10541555	Clec4a1	Mm.297275	269799	NM_199311	0.82	0.0098	0.47
10592201	Chek1	Mm.16753	12649	NM_007691	0.55	0.0098	0.47
10404026	Hist1h2af	Mm.377878	319173	NM_175661	0.52	0.0098	0.47
10404606	Ly86	Mm.2639	17084	NM_010745	0.85	0.0098	0.47
10365518	Nt5dc3	Mm.200446	103466	NM_175331	-0.46	0.0099	0.47
10498710	Bche	Mm.250719	12038	NM_009738	-0.61	0.0099	0.47
10397364	Mfsd7c	Mm.301076	217721	NM_145447	-0.52	0.0099	0.47
10588669	Rassf1	Mm.12091	56289	NM_001243748	-0.33	0.0099	0.47
10400504	Foxa1	Mm.4578	15375	NM_008259	-0.48	0.0099	0.47
10428912	Fam84b	Mm.230853	399603	NM_001162926	-0.37	0.0099	0.47
10399061	Esyt2	Mm.273755	52635	NM_028731	0.41	0.0099	0.47
10386005	Atp5f1	Mm.251152	11950	NM_009725	0.35	0.01	0.47

Supplementary Figures and Figure Legends

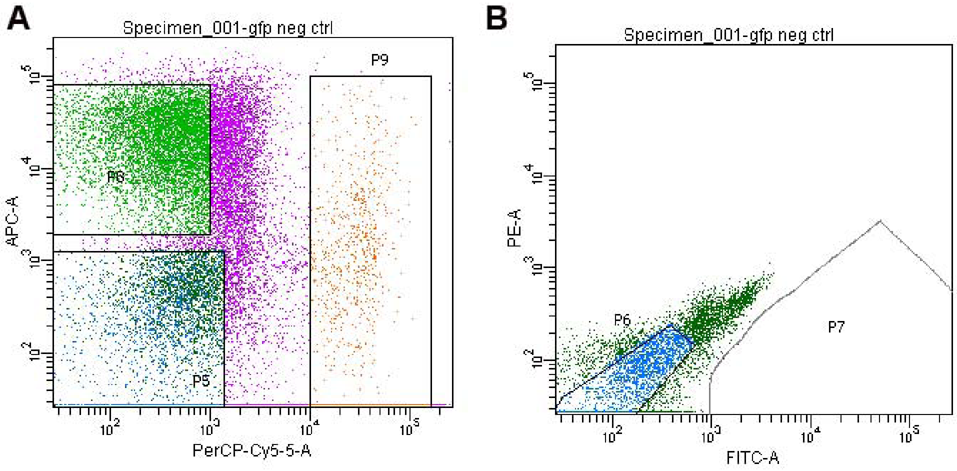

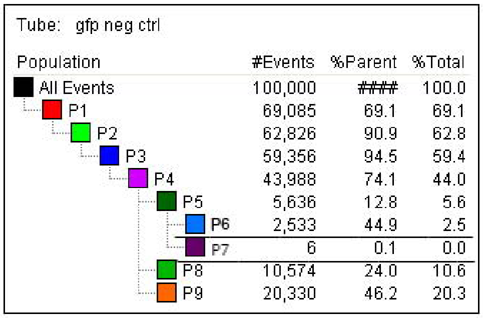
C

Tube: gfp neg ctrl

Population	#Events	%Parent	%Total
All Events	100,000	####	100.0
P1	69,085	69.1	69.1
P2	62,826	90.9	62.8
P3	59,356	94.5	59.4
P4	43,988	74.1	44.0
P5	5,636	12.8	5.6
P6	2,533	44.9	2.5
P7	6	0.1	0.0
P8	10,574	24.0	10.6
P9	20,330	46.2	20.3

Figure S1. Sorting plot of dissociated lung cells from nontransgenic mice. Lungs from wild type mice were enzymatically dissociated prior to CD45 and CD31 staining. Cells were then sorted using the established sorting algorithm for smooth muscle cell isolation from *αSMA-GFP;NG2-DsRed* mice. (**A**) $CD31^-CD45^-$ population (P5) was further sorted for GFP+ and DsRed+ cells. Using the gating developed for smooth muscle soring, no GFP^+ or $DsRed^+$ cells (in P7) were sorted from the lungs of non-transgenic mice, as shown in (**B**) and (**C**).

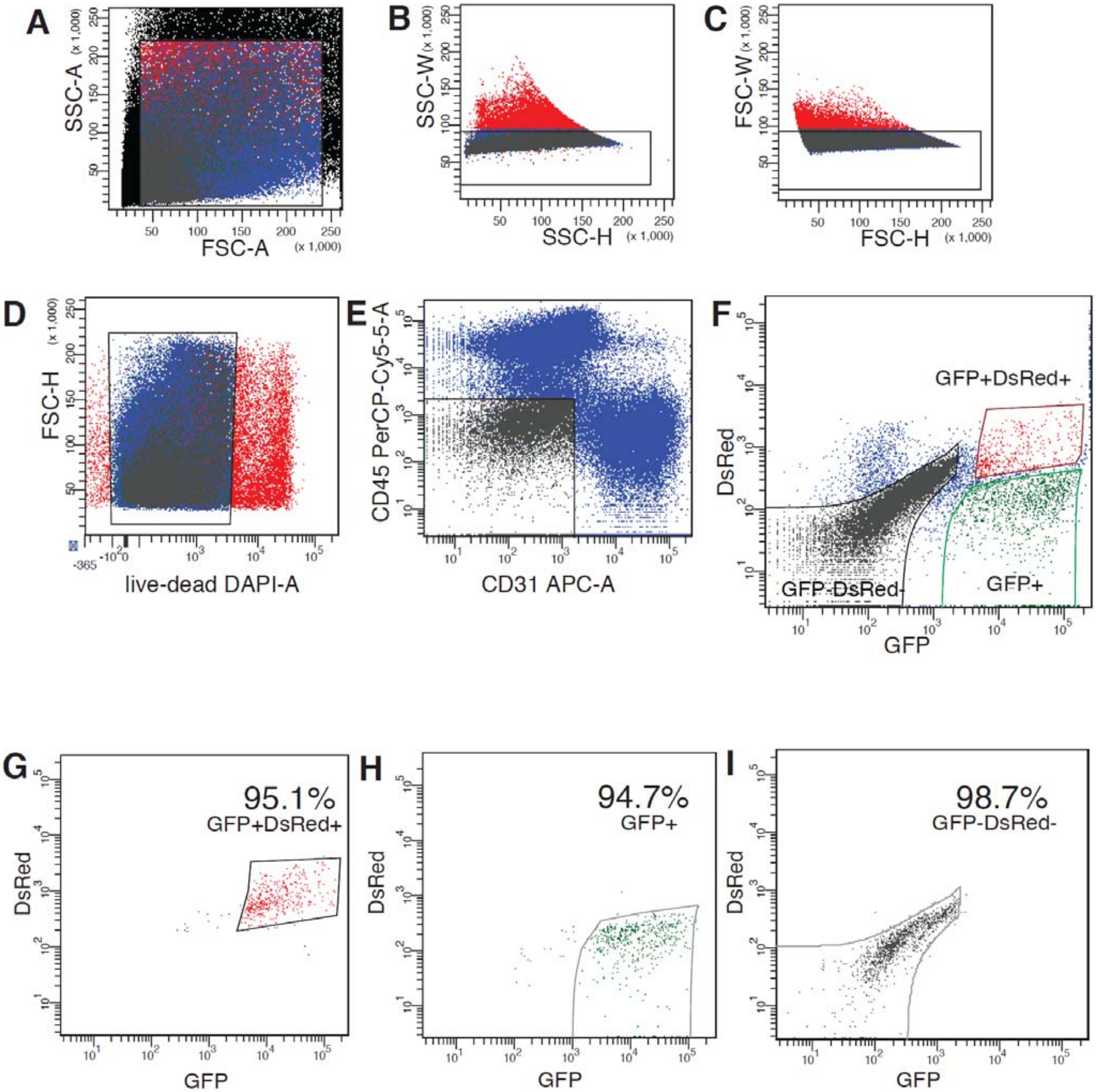

Figure S2. Post-sort purity tests for sorted hrGFP$^+$ and hrGFP$^+$DsRed$^+$ cells from the lungs of α*SMA-hrGFP+;NG2-DsRed+* mice. Cells were selected by size (**A**), doublet exclusion (**B**, **C**), and viability (**D**) followed by separation of CD45$^+$ cells and CD31$^+$ cells (**E**). The CD31$^-$CD45$^-$ population was then sorted into singly GFP$^+$, doubly GFP$^+$DsRed$^+$, and GFP$^-$DsRed$^-$ populations (**F**). Post-sort purity tests show that approximately 95% cells express appropriate markers in each population (**G, H, I**).

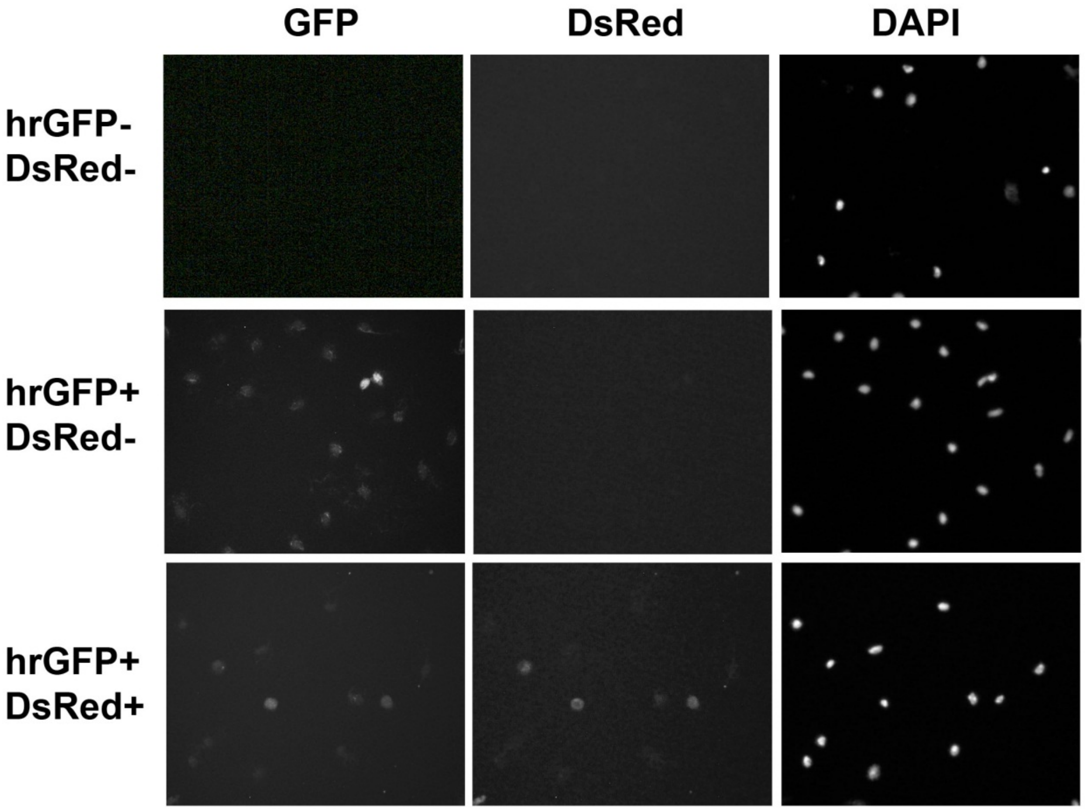

Figure S3. Examination of appropriate marker expression in sorted cell population. Sorted cells that were singly hrGFP^{+}, hrGFP^{+}DsRed^{+}, and hrGFP^{-}DsRed^{-} were collected on slides after cytospin. Cells were fixed in 4% paraformaldehyde for 5 min before DAPI nuclei staining. Cells were then examined under a fluorescent microscope for their expression of GFP and DsRed. Cells in sorted hrGFP^{-}DsRed^{-} population did not express GFP or DsRed. A large majority of cells in sorted singly hrGFP^{+} population expressed only GFP but not DsRed. In addition, most of cells in sorted hrGFP^{+}DsRed^{+} population expressed both GFP and DsRed. These results confirmed the fidelity of our sorting algorithm.

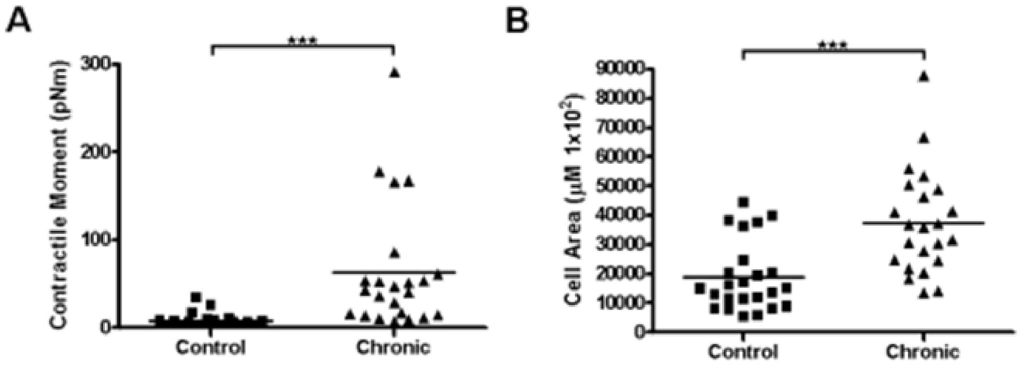

Figure S4. Characterization of physical properties of BSMCs from a mouse model of chronic asthma. (**A**) Contractile moment measurement of individual control BSMCs and BSMCs from a mouse model of chronic asthma. The line represents the mean of individual measurements of control BSMCs (●; n=20) and BSMCs from the chronic model (▲; n=23). (**B**) Measurement of cell size of control BSMCs (●) and BSMCs from the chronic model (▲; n=23) BSMCs. The line represents the mean of individual measurements. ***P<0.0001.

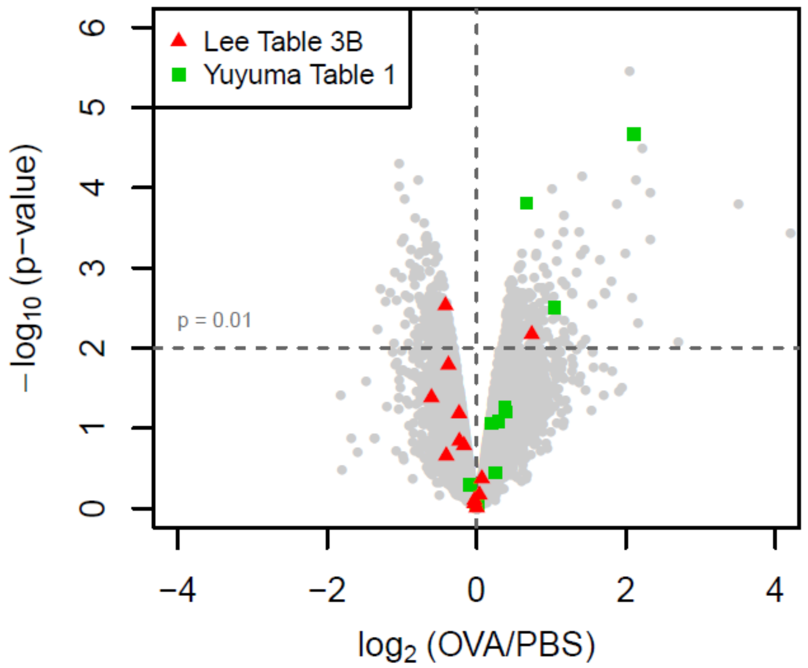

Figure S5. The concordance of our BSMCs array results with sets of genes identified as differentially expressed by previous asthma-related studies [1, 2], as assessed by the gene set analysis method ROAST [3]. The volcano plot displays $\log_2$(fold change in OVA vs. PBS) (LFC) against $-\log_{10}$ (p-value from t-test) for each of the ~20,000 genes on the arrays. Mouse orthologs of genes from table 3B in Lee et al [1] (genes down-regulated by IL-13 in human airway epithelial cells) are shown as red triangles, and tend to be down-regulated (LFC < 0) in our BSMC arrays with OVA sensitized mice and saline control mice. Mouse orthologs of genes from table 1 of Yuyuma et al. [2] (genes induced by IL-4 and IL-13 in human bronchial epithelial cells) are shown as green squares, and tend to be up-regulated (LFC >0) in our BSMC arrays. Genes above the dashed horizontal line have $p < 0.01$.

.

References

1. Lee JH, Kaminski N, Dolganov G, Grunig G, Koth L, et al. (2001) Interleukin-13 induces dramatically different transcriptional programs in three human airway cell types. *Am J Respir Cell Mol Biol* 25: 474-485.
2. Yuyama N, Davies DE, Akaiwa M, Matsui K, Hamasaki Y, et al. (2002) Analysis of novel disease-related genes in bronchial asthma. Cytokine 19: 287-296.
3. Wu D, Lim E, Vaillant F, Asselin-Labat ML, Visvader JE, et al. (2010) ROAST: rotation gene set tests for complex microarray experiments. Bioinformatics 26: 2176-2182.
